# A review of reviews on principles, strategies, outcomes and impacts of research partnerships approaches: a first step in synthesising the research partnership literature

**DOI:** 10.1186/s12961-020-0544-9

**Published:** 2020-05-25

**Authors:** F. Hoekstra, K. J. Mrklas, M. Khan, R. C. McKay, M. Vis-Dunbar, K. M. Sibley, T. Nguyen, I. D. Graham, SCI Guiding Principles Consensus Panel, Kim Anderson, Hugh Anton, Peter Athanasopoulos, John Chernesky, Susan Forwell, Jocelyn Maffin, Kathleen Martin Ginis, Christopher B. McBride, Ben Mortenson, Rhonda Willms, H. L. Gainforth

**Affiliations:** 1 School of Health & Exercise Sciences, University of British Columbia, Kelowna, Canada; 2 International Collaboration on Repair Discoveries (ICORD), University of British Columbia, Vancouver, Canada; 3 Strategic Clinical Networks™, System Innovation and Programs, Alberta Health Services, Calgary, Alberta Canada; 4 Department of Community Health Sciences, Cumming School of Medicine, University of Calgary, Calgary, Alberta Canada; 5 Department of Community Health Sciences, Max Rady College of Medicine, University of Manitoba, Winnipeg, Manitoba Canada; 6 Library, University of British Columbia Okanagan, Kelowna, British Columbia Canada; 7 George & Fay Yee Centre for Healthcare Innovation, University of Manitoba, Winnipeg, Manitoba Canada; 8 School of Epidemiology and Public Health, Faculty of Medicine, University of Ottawa, Ottawa, Ontario Canada; 9 CanChild Centre for Childhood Disability Research, Faculty of Health Sciences, McMaster University, Hamilton, Ontario Canada; 10 Clinical Epidemiology Program, Ottawa Hospital Research Institute, Ottawa, Ontario Canada; 11 School of Epidemiology and Public Health, University of Ottawa, Ottawa, Ontario Canada

**Keywords:** Collaborative research partnerships, Integrated knowledge translation, Community-based participatory research, Stakeholder engagement, Research principles and strategies, Research outcomes and impact, Knowledge syntheses

## Abstract

**Background:**

Conducting research in partnership with stakeholders (e.g. policy-makers, practitioners, organisations, patients) is a promising and popular approach to improving the implementation of research findings in policy and practice. This study aimed to identify the principles, strategies, outcomes and impacts reported in different types of reviews of research partnerships in order to obtain a better understanding of the scope of the research partnership literature.

**Methods:**

This review of reviews is part of a Coordinated Multicenter Team approach to synthesise the research partnership literature with five conceptually linked literature reviews. The main research question was ‘What principles, strategies, outcomes and impacts are reported in different types of research partnership approaches?’. We included articles describing a literature review of research partnerships using a systematic search strategy. We used an adapted version of the Revised Assessment of Multiple Systematic Reviews tool to assess quality. Nine electronic databases were searched from inception to April 2018. Principles, strategies, outcomes and impacts were extracted from the included reviews and analysed using direct content analysis.

**Results:**

We included 86 reviews using terms describing several research partnership approaches (e.g. community-based participatory research, participatory research, integrated knowledge translation). After the analyses, we synthesised 17 overarching principles and 11 overarching strategies and grouped them into one of the following subcategories: relationship between partners; co-production of knowledge; meaningful stakeholder engagement; capacity-building, support and resources; communication process; and ethical issues related to the collaborative research activities. Similarly, we synthesised 20 overarching outcomes and impacts on researchers, stakeholders, the community or society, and the research process.

**Conclusions:**

This review of reviews is the first that presents overarching principles, strategies, outcomes and impacts of research partnerships. This review is unique in scope as we synthesised literature across multiple research areas, involving different stakeholder groups. Our findings can be used as a first step to guide the initiation and maintenance of research partnerships and to create a classification system of the key domains of research partnerships, which may improve reporting consistency in the research partnership literature.

**Trial registration:**

This study is registered via Open Science Framework: 10.17605/OSF.IO/GVR7Y.

## Background

Increasingly, research partnerships in which researchers and stakeholders work together on a research project are becoming a widely accepted, and sometimes mandated, approach to implementation [1–5]. These partnerships aim to shift the research paradigm from one in which the researcher is the sole expert to one in which researchers and stakeholders co-lead research activities and collectively apply their expertise, knowledge and skills within a team [6]. While these research partnerships are a broadly accepted tenet of knowledge translation [7], there is limited literature describing the optimal processes (i.e. principles, strategies) for research coproduction and limited empirical evidence supporting the perceived outcomes or impacts of working in partnership.

The variability in research partnership approaches and terminologies (e.g. community-based participatory research (CBPR), participatory action research (PAR), integrated knowledge translation (IKT)) across multiple research fields present substantial challenges for syntheses in the field of research partnerships, including complexities arising from diverse definitions, conceptual similarities/differences, evidence volume and dispersion, logistics/resource and feasibility issues [1, 8–10]. Syntheses of various partnership approaches from different research fields are needed to develop an understanding of the literature, learn from others’ successes and challenges, and to advance the science of research partnerships and implementation [11]. To address these challenges, our team developed a collaborative review approach (i.e. Coordinated Multicenter Team) to reviewing and synthesising research partnership literature [11]. This standardised approach is described elsewhere [11] and aims to conduct five conceptually linked literature reviews focusing on research partnerships. To address the gaps in the research partnership literature [7, 8], the approach is guided by a consensus-driven conceptual framework and is focused on four key domains of research partnership – principles, strategies, outcomes and impacts. Additional file 1: Appendix 1 presents the framework and related definitions.

This review presents findings from the first step in our Coordinated Multicenter Team approach – a review of reviews on key domains of research partnerships. In accordance with our conceptual framework, this review of reviews identifies the principles, strategies, outcomes and impacts reported in different types of research partnership approaches in order to gain a better understanding of the scope of the research partnership literature. In particular, this review (1) provides an overview of terms, definitions and descriptions used in the research partnership literature, and (2) synthesises overarching principles, strategies, outcomes and impacts of research partnership approaches.

As the review of reviews primarily aimed to guide our next steps in synthesising the research partnership literature (i.e. scoping reviews and umbrella reviews), this review of reviews did not aim to provide a comprehensive overview of the research partnership literature.

## Methods

### Study protocol and registration

This paper describes a review of reviews focusing on four key domains of research partnerships – principles, strategies, outcomes and impacts (Additional file 1: Appendix 1). The study was guided by the Preferred Reporting Items for Systematic Reviews and Meta-Analyses (PRISMA) [12] and by Pollock et al. [13] for the conduct of overviews of reviews. Additional file 1: Appendix 2 presents details about adherence to the PRISMA guidelines. The published protocol [11] was registered in Open Science Framework (OSF) [14, 15]. Protocol deviations are reported on OSF [16].

### Search strategy and eligibility criteria

The following data sources were searched by academic librarians (MVD, CN): Medline, Embase, CINAHL, PsycINFO, Eric, Education Source, Social Services Abstracts, Sociological Abstracts, Sociology Database, Applied Social Sciences Index and Abstracts, Web of Science Core Collection and JSTOR. The health databases (Medline, Embase, CINAHL, PsycInfo) were searched from inception to January 2018 and updated in April 2018. The other databases were searched from inception to April 2018. The search strategy identified reviews on the following concepts: partnership research, participatory research, knowledge translation and knowledge transfer. As we did not aim to provide a comprehensive overview of the research partnership literature, we opted for a high-level search strategy to capture global terms related to partnership, participatory and community-focused research. Our working hypothesis was that key reviews on research partnership approaches would use standardised, aggregated expressions and terminology to describe the research processes being reviewed [11]. As described in our protocol paper [11], the findings from this review were to be used to develop a more comprehensive search strategy for our subsequent scoping reviews that leverages both standardised terms across disciplines as well as natural language expressions of partnership research. In collaboration with academic librarians (MVD, CJN), we decided to start our collaborative review process (i.e. this review of reviews) with a high level search strategy and subsequently develop a refined and more comprehensive search strategy for our scoping reviews aligned with our general collaborative focus on maximising search strategy efficiencies and optimising research quality [11].

Final search strategies for each database are available on OSF (OSF – Table I). In addition to the database search, reference lists of included reviews were scanned to identify relevant reviews.

We included reviews that described a literature review on how research partnerships work (i.e. principles or strategies) or described the outcomes or impacts of research partnerships. Table 1 presents the inclusion and exclusion criteria.

**Table 1:** Inclusion and exclusion criteria

Topic	Inclusion criteria	Exclusion criteria
**Scope**	- The aim/objective/purpose/research question of the literature review should focus (partly) on research partnership (e.g. how partnerships work; what are the outcomes or impacts) - The paper describes a literature review on how research partnerships work (i.e. principles or strategies) OR the paper describes a literature review on outcomes or impacts of research partnerships - The literature review included studies that described or evaluated the research partnership OR described or evaluated the collaborative research activity OR described or evaluated methods or tools to study partnerships or collaborative research activity	- The paper used/applied a research partnership approach without studying it - The paper concluded that research partnerships are relevant/useful without studying it - The paper describes a literature review on knowledge translation and/or knowledge mobilisation without a focus on research partnership - The paper does not include any extractable data related to principles, strategies, outcomes or impacts
**Definition**	- The paper meets our definition of research partnership: ◦ Research partnership is defined as “*individuals, groups or organisations engaged in collaborative research activity involving at least one researcher (e.g., individual affiliated with an academic institution), and any stakeholder (e.g., decision or policy-maker, healthcare administrator or leader, community agency, charities, network, patients etc.)*” [2, 11] - The paper includes a definition or description of the research partnership approach	- The paper does not meet our definition of research partnership. Examples include: ◦ A researcher is not part of the partnership (e.g. physician–patient partnership; student–teacher partnership) ◦ A stakeholder is not part of the partnership (e.g. partnership between researchers from different disciplines or different countries) - The paper focused on public–private partnerships or university–industry partnerships - The paper does not describe or define the research partnership approach
**Design and search strategy**	- The paper describes a literature overview of research partnerships - The paper used a systematic search (provided a general description of their search strategy in terms of their search terms, eligibility criteria and databases that are searched) of the literature	- The paper describes a review of a method or tool instead of a literature overview - The paper combined the literature study with another study design (e.g. case study) without making a distinction between the results derived from the literature review and the other data source - The paper searched only grey literature; the paper did not search electronic databases (e.g. ERIC, Medline, PsycInfo)
**Language**	- The paper is published in English language	- The paper is not published in English language

*Notes:* Additional file 1: Appendix 1 presents our guiding framework and related key definitions of the research partnership domains (principles, strategies, outcomes, impacts)

### Engagement of stakeholders in the review

A steering committee, consisting of a group of stakeholders interested in developing guiding principles for conducting and disseminating research in partnership with people with spinal cord injury (SCI), was established (the SCI Guiding Principles Consensus Panel). The panel members’ names, organisations and roles are described in Additional file 1: Appendix 3, including people with lived experience of SCI, decision-makers, healthcare professionals, representatives from community organisations and researchers. For this review of reviews, members were engaged in three key research activities, namely (1) conceptual design and formulation of the research questions; (2) preparation of data extraction forms; and (3) data analysis, interpretation and dissemination of results.

Additional file 1: Appendix 4 provides an overview of our participatory (IKT) approach, including the collaborative research activities, associated dates, topics discussed, stakeholders’ concerns and suggestions, and our responses.

### Screening process

Search results were exported to Endnote X.7.5.3 and de-duplication was conducted following the steps described by Bramer et al. [17]. The results were exported, managed and analysed using a combination of Rayyan [18] and Excel.

The screening process was executed in three phases – title, abstract and full text. First, titles of all citations were independently screened by at least two team members (FH, KJM, MK). Only citations excluded by two team members were excluded in this screening phase. Second, title and abstracts were independently screened by two team members (FH and KJM, FH and MK, FH and RM) using the abstract-level eligibility criteria. Reliability between each pair of screeners (FH and KJM, FH and MK, FH and RM) was calculated using Cohen’s Kappa statistic during this and full text screening [19]. All discrepancies between coders were discussed and resolved through a consensus discussion. Third, full-texts were screened independently by two team members (FH and MK, FH and RM) using inclusion criteria described in Table 1. Discrepancies between coders were discussed and resolved. If necessary, a third team member (MK or RM) was contacted for a final decision.

### Data extraction and analyses

The first author (FH) and research assistants (PS, DS, KW) extracted general review characteristics and partnership characteristics using an online data extraction form. Review characteristics were extracted, including the year of publication, country of first author, title, study aims, research area, research population and type of literature review. Extracted partnership characteristics included key terms used to describe the research partnership approach (e.g. CBPR, PAR, IKT), definition or description of the research partnership, and partnerships’ members. After data extraction, one researcher (FH) organised the information related to the research area and research population. The findings were then discussed and refined during a meeting with other researchers (KJM, MK, KS, TN, HG). Key characteristics of each review were exported to an Excel sheet and published on OSF – Table III.

Two researchers worked together to extract the data related to the key domains (principles/strategies: FH and RM; outcomes/impacts: FH and MK) using definitions described in Additional file 1: Appendix 1. We used an iterative extraction and analysis process, guided by direct content analysis [20], consisting of the following steps:

- *Development of coding manual.* We developed three coding manuals (principles, strategies, outcomes/impacts) using the extracted information of 8 randomly selected reviews (~10%). In contrast to our protocol, a single coding manual was created by combining outcomes and impacts. A combined manual was deemed a better fit given the lack of clear differentiation, use of terms, and reporting of outcomes and impacts in eligible papers. The three manuals were created iteratively. Two researchers (FH, RM or MK) extracted the data independently and, after each review, both researchers discussed the extracted data and resolved disagreements. One these researchers then performed data extraction of the remaining 78 reviews using the established coding manual.
- *First analysis round*. After data was extracted from 66 included reviews (~80%), one researcher (FH) conducted a first analysis, in which codes were removed, refined and/or grouped together. All decisions were reviewed by another researcher (RM, MK or HG), and disagreements were discussed and resolved. We then grouped codes into (sub)categories. This process resulted in three Excel sheets listing principles, strategies and outcomes/impacts. Researchers (FH, MK) and research assistants (FR, MK) completed these Excel sheets based on the extracted data of the reviews.
- *Second analysis round*. After we extracted data from all reviews, one researcher (FH) conducted a second analysis, in which codes were again removed, refined and/or grouped together, and the data was re-organised. All decisions were reviewed by another researcher (RM, MK, HG) and disagreements were discussed and resolved.
- *Final analysis round*. During the final analysis step, we synthesised the data into workable sets of overarching principles, strategies and outcomes/impacts. The project leads (FH, HG) synthesised the lists of principles and strategies into two sets of overarching principles and strategies. One project lead (FH) synthesised the list of outcomes/impacts into a set of overarching outcomes/impacts. These overarching findings were then discussed and refined during a meeting with other researchers (MK, KJM, KMS, TN). The overarching principles and strategies were also discussed and refined after a meeting with the steering committee (Additional file 1: Appendix 3 and 4). Next, one researcher (IG), who is an IKT and KT expert, reviewed the overarching findings and provided critical feedback on language and clarifications. The project leads (FH, HG) then refined the overarching findings. Finally, all co-authors and panel members reviewed and approved the final sets of overarching principles, strategies and outcomes/impacts.

### Methodological quality appraisal and risk of bias

The Revised Assessment of Multiple Systematic Reviews (R-AMSTAR) survey [21] was completed independently and in duplicate by two research assistants (CM, KW, PS, DW, MB, FR, MK, KL). If the total scores varied between assessors by more than 5 points (11%), disagreements were discussed and/or resolved by another researcher (FH, CM). For each review, a total mean score was calculated based on ratings from the two assessors. Following previously described procedures [21], the total mean scores were converted into percentiles and grouped into four grades (A, B, C, D): ≥90, 80–89, 70–79, and ≤69 percentiles. Grade A includes reviews with the highest rated quality and grade D includes reviews with the lowest rated quality. Total mean scores were used to identify whether the quality of the review was related to publication year, research area and/or type of review.

Given that the goal of this review was to provide an overview of research partnership literature and its key domains, we did not use the quality assessment scores to synthesise our findings on overarching principles, strategies and outcomes/impacts. Similarly, we did not include a systematic risk of bias assessment. Potential risks of biased results are described in the Discussion section.

## Results

### Literature search

**Fig. 1:**
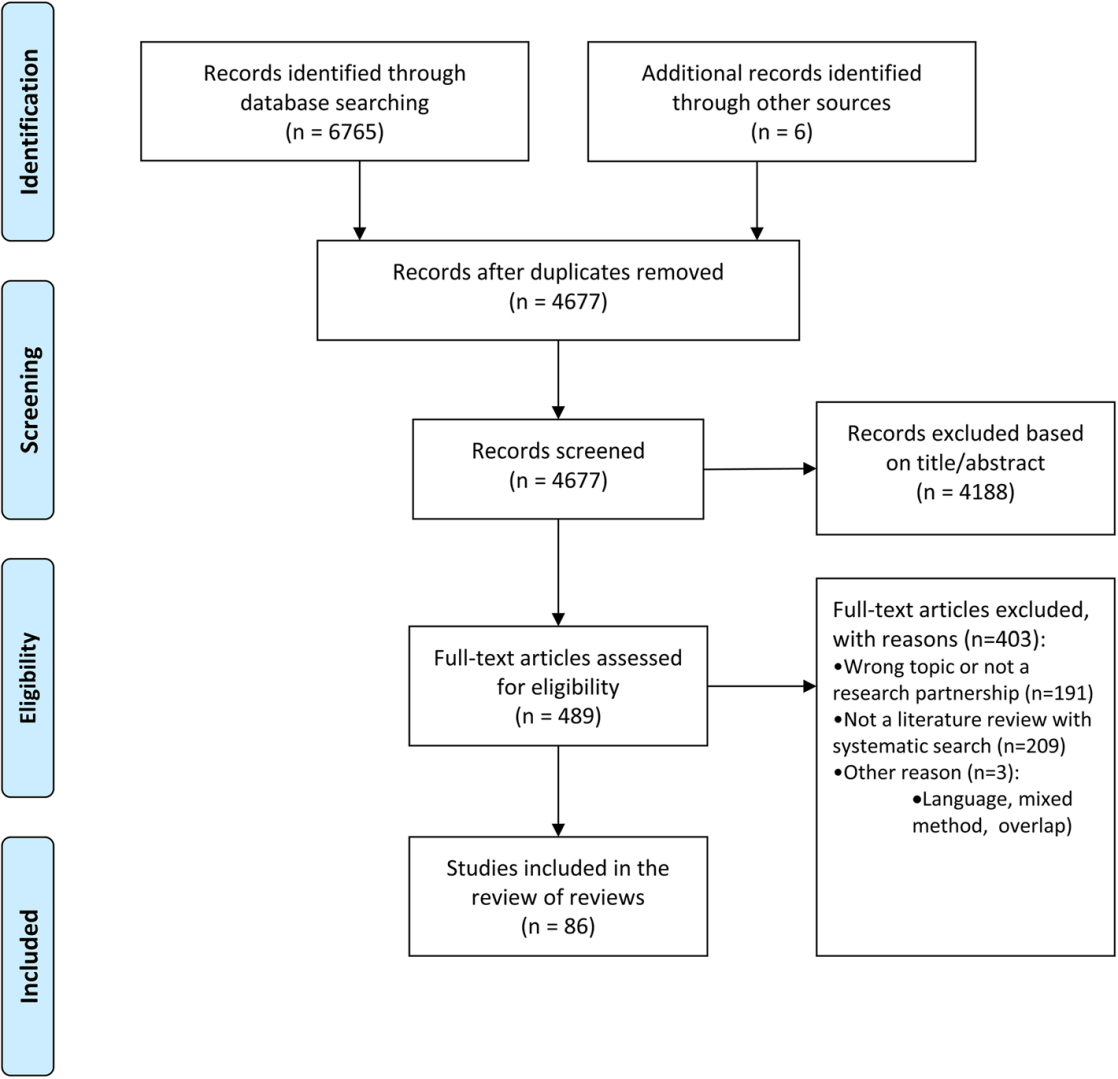
The PRISMA flowchart

The literature search provided a total of 4677 unique citations (Fig. 1). After screening titles and abstracts, 4188 articles were excluded. The full texts of the remaining 489 papers were retrieved and reviewed. A total of 86 reviews were included in this review of reviews. Agreement between screeners for abstracts was considered as “substantial” for abstract screening (Mean Cohen’s Kappa for each of the three screening pairs: 0.71, 0.67, 0.63) and full-text screening (Mean Cohen’s Kappa for each of the two screening pairs: 0.71, 0.74). References of included reviews are presented in Additional file 1: Appendix 5, and the list of excluded papers is published on OSF [16].

### Review characteristics and application areas

Table 2 provides a summary of the study characteristics of the included reviews. Fourteen (16%) reviews were published between the years 2004 and 2011, 48 (55%) reviews were published between the years 2012 and 2016, and the remaining 24 (28%) reviews were published in 2017 or 2018.

**Table 2:** Review characteristics of included reviews (*n*=86)

First author	Year	Country	Sub-area	Research population	Partnership term	Type of review^b^	Included documents^a^	Quality score^c^	Grade^d^
**Population health (*n*=34)**									
Andrews [22]	2012	USA	Smoking cessation	Marginalized communities	CBPR	Integrative review	11	24.5	D
Bach [23]	2017	Germany	Epidemiology	General population	PR	Scoping review	102	22	D
Blair [24]	2009	USA	Gerontology	Elderly	PAR	Literature review	13	25	D
Brizay [25]	2015	Switzerland	HIV-related research	People with HIV	Combination	Systematic review	149	23.5	D
Catalani [26]	2010	USA	Not specified	Not specified	CBPR	Literature review	37	22.5	D
Chen [27]	2010	USA	Not specified	General population	CBPR	Systematic review	101	24	D
Cook [28]	2008	USA	Environmental health	General population	CBPR	Systematic review	36	25.5	C
Commodore [29]	2017	USA	Environmental research	General population	CBPR	Literature review	33	18	D
Coughlin [30]	2017	USA	Obesity prevention	Ethnic minority groups	CBPR	Literature review	16	21	D
Cyril [31]	2015	Australia	Not specified	Disadvantaged populations	Other	Systematic review	24	27.5	B
Dempsey [32]	2014	USA	Mental Health	People with mental illness/ disorder	CBPR	Systematic Review	38	21.5	D
Eyles [33]	2016	New Zealand	mHealth interventions	Ethnic minority groups	CBPR	Literature review	9	19.5	D
Forsythe [34]	2014	USA	Not specified	Patients with rare disease	Other	Systematic Review	35	29	A
Harrop [35]	2012	USA	Cancer research	General population	CBPR	Literature review^e^	9	19	D
Hergenrater [36]	2009	USA	Not specified	Not specified	CBPR	Qualitative review	31	18.5	D
Hubbard [37]	2007	UK	Cancer research	People affected by cancer	Other	Literature review	131	19.5	D
Jivray [38]	2014	Canada	Mental Health	People with mental illness/ disorder	PR	Scoping review	7	19	D
Joss [39]	2016	Australia	Disability Research	People with disability	Other	Scoping review	27	21.1	D
Krishnaswami [40]	2012	USA	Health promotion	Children and youth	Other	Systematic review	16	33	A
Lesser [41]	2007	USA	Not specified	Vulnerable populations	Other	Literature review	25	15	D
McVicar [42]	2013	UK	Workplace stress	General population	PAR	Scoping review	11	15.5	D
Miller [43]	2012	Australia	Cancer research	Indigenous populations	Other	Narrative review	37	23.5	D
Nitsch [44]	2013	Austria	Health promotion	General population	Other	Literature review	42	22	D
Orlowski [45]	2015	Australia	Mental Health	Children and youth	PR	Systematic review	17	28	B
Portillo [46]	2004	USA	Nursing	General population	CBPR	Literature review	30	16.5	D
Salimi [47]	2012	Iran	Not specified	General population	CBPR	Systematic review	8	28	B
Salsberg [48]	2015	Canada	Not specified	Not specified	PR	Critical review	54	19.5	D
Snijder [49]	2015	Australia	Not specified	Indigenous populations	Other	Systematic review	31	31	A
Speights [50]	2017	USA	Not specified	Ethnic minority groups	CBPR	Narrative review	28	18.5	D
Stacciarini [51]	2011	USA	Mental Health	Minority populations	CPBR	Integrative review	20	18.5	D
Stacciarini [52]	2009	USA	Mental Health	Ethnic minority groups	CBPR	Literature review	42	22	D
Vaughn [53]	2013	USA	Pediatric	Children and youth	CBPR	Literature review	34	18	D
Vaughn [54]	2017	USA	Not specified	Ethnic minority groups	CBPR	Literature review	161	21.5	D
Wine [55]	2017	Canada	Environmental research	Not specified	Other	Scoping review	45	25.5	C
**Health services (*n*=25)**									
Adebayo [56]	2017	USA	Not specified	Vulnerable populations	Other	Systematic review	32	23	D
Backhouse [57]	2016	UK	Not specified	Elderly	PPI	Systematic review	19	27.5	B
Baines [58]	2018	UK	Not specified	Not specified	PPI	Systematic review of reviews^e^	90	20	D
Brear [59]	2017	Australia	Resource-constrained countries	Not specified	PR	Scoping review	85	21	D
Bush [60]	2017	Canada	Health organizations	Not specified	PR	Systematic mixed studies review	177	27.5	B
Camden [1]	2015	Canada	Rehabilitation care	Rehabilitation patients	Other	Scoping review	19	25.5	C
Concannan [61]	2014	USA	Not specified	Not specified	Other	Literature review	157	21	D
DeLasNueces [62]	2012	USA	Clinical trials	Ethnic minority groups	CBPR	systematic review	19	24	D
Domecq [63]	2014	USA	Not specified	Patients	Other	Systematic review	142	31	A
Ehde [64]	2013	USA	Rehabilitation care	Rehabilitation patients	PAR	Literature review^e^	5	22	D
Frankena [65]	2015	The Netherlands	Not specified	People with intellectual disabilities	Other	Literature review	26	24	D
Gagliardi [66]	2016	Canada	Not specified	Not specified	IKT	Scoping review	13	28.5	B
Ganann [67]	2013	Canada	Not specified	Ethnic minority groups	PAR	Literature review	n.c.	15	D
Greenhalgh [68]	2016	UK	Not specified	Not specified	Other	Literature review^e^	110	18	D
Jagosh [4]	2012	Canada	Not specified	Not specified	PR	Realist review	276	26	C
Manafo [69]	2018	Canada	Not specified	Not specified	PPI	Rapid review	70	28.5	B
Nilsen [70]	2013	UK	Not specified	Not specified	Other	Systematic review	6	40	A
Noh [71]	2016	USA	Palliative Care	People using palliative care services	CBPR	Literature review	18	21.5	D
Nwanyanwu [72]	2017	USA	Ophthalmology	Not specified	Other	Systematic review	18	22	D
Shen [73]	2012	Canada	Not specified	Parents	Other	Scoping review	10	33	A
Smith [74]	2008	UK	Nursing	Service users	Other	Literature review	n.c.	17	D
Soh [75]	2011	Malaysia	Intensive care	Service users	AR	Systematic review	21	26	C
Tricco [76]	2018	Canada	Not specified	Not specified	Other	Scoping review	91	31	A
Vaughn [77]	2017	USA	Not specified	A variation of patient groups	Other	Literature review	103	21.5	D
Vollmn [78]	2017	UK	Forensic mental health services	Service users	Other	Rapid review	23	25	D
**Health and social science (*n*=16)**									
Adams [79]	2012	Australia	Not specified	Indigenous populations	Other	Mini-literature review^e^	20	20	D
Bailey [57]	2014	UK	Not specified	Children and youth	PPI	Systematic review	22	26	C
Brett [80]	2014	UK	Not specified	Not specified	PPI	Systematic review	65	27	C
Brett [81]	2012	UK	Not specified	Not specified	PPI	Systematic review	55	26	B
Carter [82]	2015	USA	Family planning	General population	Other	Systematic review	11	22	D
Crabtree [83]	2013	USA	Natural disaster	Vulnerable populations	CBPR	Systematic review^f^	14	22	D
Dawson [84]	2017	UK	Not specified	Ethnic minority groups	PPI	Systematic review	69	26.5	C
DiLorito [85]	2016	UK	Not specified	Elderly/ Dementia	PPI	Literature review	7	23	D
DiLorito [86]	2017	UK	Co-research process	People with intellectual disabilities	Other	Systematic review	13	29.5	A
Drahota [2]	2016	USA	Not specified	Not specified	Other	Systematic review^e^	50	29	A
Haijes [87]	2016	The Netherlands	Pediatric	Children and youth	PR	Systematic review	24	25.5	C
Jacquez [88]	2013	USA	Not specified	Children and youth	CBPR	Literature review	385	23	D
Ragavan [89]	2018	USA	Domestic Violence	Domestic Violence survivors	CBPR	Systematic review	20	23.5	D
Strnadov [90]	2017	Australia	Inclusive research	People with intellectual disabilities	Inclusive research	Literature review	52	22	D
Trembley [91]	2017	Canada	Not specified	Not specified	CBPR	Framework synthesis review	8	26	C
Viswanathan [92]	2004	USA	Not specified	Not specified	CBPR	Systematic review	123	32.5	A
**Research ethics (*n*=7)**									
Coons [93]	2013	Canada	Not specified	People with intellectual disabilities	PAR	Literature review	n.c.	13	D
Fouche [94]	2017	New Zealand	Not specified	Not specified	AR	Literature review	39	17.5	D
Kwan [95]	2018	Canada	Not specified	Not specified	CBPR	Narrative review	40	13.5	D
Mikesell [96]	2013	USA	Not specified	Not specified	CBPR	Systematic review	57	19.5	D
Souleymanov [97]	2016	Canada	Not specified	People who use drugs	CBPR	Scoping review	25	24	D
Tamariz [98]	2015	USA	Not specified	Not specified	CBPR	Literature review	10	26.5	C
Wilson [99]	2018	Australia	Not specified	Not specified	CBPR	Literature review	48	24	D
**Biomedical research (*n*=4)**									
Shippee [100]	2013	USA	Not specified	Not specified	Other	Systematic review	41	20.5	D
Tindana [101]	2015	Ghana	Genomic studies	Not specified	Other	Literature review	38	20.5	D
Young [102]	2017	Canada	Orphan drugs	Patient with rare diseases	Other	Scoping review	150	25	D
Yusuf [103]	2015	Canada	Biomarker discovery	People with mental illness/ disorder	CBPR	Scoping review	7	17	D
**Total (86)**							**4395**^**b**^		

*Notes:* Full references of the reviews are included in supplementary file. Reviews are grouped into one of the five main areas: population health, health services, health and social sciences, research ethics, or biomedical research. If applicable, identified sub-area were listed under ‘sub-area’. The research population refers to the population that the research was focusing on (i.e. this may differ from the members of the partnerships). Not specified indicated that no specific sub-area or research population was identified

*n.c.* not clear, *CBPR* Community-based participatory research, *PAR* Participatory Action Research, *PR* Participatory Research, *IKT* Integrated Knowledge Translation, *AR* Action Research, *PPI* Patient and Public Involvement

^a^The review type of is the review type (term) reported by the authors of the review. These labels should be interpreted with caution, as authors may have used different definitions

^b^The number of included documents is the total number of included studies and documents from grey literature search. The total number of documents (n=4395) may include duplicates, as we did not take into account that different reviews have included the same primary studies

^c^The mean of the total scores on the R-AMSTAR assessed by two independent assessors

^d^The percentile grades: grade A: ≥90%ile, grade B: 80–89%ile, grade C: 70–79%ile, grade D: ≤69%ile, in which grade A represents reviews with the highest quality and grade D represents reviews with the lowest quality

^e^A mixed-method study

^f^A thesis chapter and not published in a peer-reviewed journal

The majority of the reviews were reported as systematic reviews (*n* = 31), literature reviews (*n* = 27) or scoping reviews (*n* = 14). Other review types included narrative reviews (*n* = 3), an integrative review (*n* = 2), rapid reviews (*n* = 2), a critical review (*n* = 1), a framework synthesis review (*n* = 1), a mini-literature review (*n* = 1), a qualitative review (*n* = 1), a realist review (*n* = 1), a systematic review of reviews (*n* = 1) and a systematic mixed studies review (*n* = 1). Six reviews [2, 35, 58, 64, 68, 79] used a mixed-methods approach, indicating that the literature review was combined with another type of study design (e.g. interview study, case study, Delphi study).

Reviews were published by first authors from the United States (*n* = 36), Canada (*n* = 17), the United Kingdom (*n* = 14), Australia (*n* = 9), New Zealand (*n* = 2), the Netherlands (*n* = 2), Austria (*n* = 1), Iran (*n* = 1), Germany (*n* = 1), Ghana (*n* = 1), Malaysia (*n* = 1) and Switzerland (*n* = 1). Reviews were conducted in various research areas: population health (*n* = 34), health services (*n* = 25), health and social sciences (*n* = 16), research ethics (*n* = 7) and biomedical research (*n* = 4). Within the population health domain, mental health (*n* = 5), environmental research (*n* = 3) and cancer research (*n* = 3) were the most mentioned subareas. A selection of reviews (*n* = 25) focused on research partnerships with specific groups of stakeholders, such as Indigenous and ethnic minority populations (*n* = 4) [50, 54, 67, 79], children and youth (*n* = 3) [87, 88, 104], elderly (*n* = 3) [24, 57, 85], organisations, managers, decision- or policy-makers (*n* = 3) [56, 66, 76], people with intellectual disabilities (*n* = 3) [65, 86, 90], people with mental illness (*n* = 3), and other vulnerable populations (*n* = 5).

### Quality assessment

The median of the R-AMSTAR total scores was 23.00 (IQR, 20–26) (Table 2). The majority of the reviews were classified in the low or moderate percentile grades – grade D: *n* = 57 (66%), grade C: *n* = 11 (13%), grade B: *n* = 8 (9%). Ten (12%) reviews were classified in the highest grade (grade A: *n* = 10). The R-AMSTAR scores of reviews on research ethics were the lowest compared to reviews in other areas (research ethics, 19.50; biomedical, 20.50; population health, 21.75; health services, 24.00; health and social science, 25.75). The R-AMSTAR scores were highest among systematic reviews compared to the other review types (systematic reviews, 26.00; scoping reviews, 24.00; and literature reviews, 21.50).

### Nature of stakeholder engagement

In 18 of the 86 reviews (21%) [1, 34, 47, 49, 53, 54, 59, 61, 62, 65, 66, 73, 84, 86, 88, 89, 92, 100, 105] detailed information could be extracted on the engagement of stakeholders in different phases of the research process (e.g. planning phase, conducting research, dissemination of findings) (Table 3). Without checking for potential overlap in primary studies included in these reviews, the 18 reviews covered ~870 primary studies. This set of reviews showed that stakeholders were most frequently reported to be engaged in identifying research questions (423/787, ~54%), followed by developing study design and/or methods (393/831, 47%), data collection (374/824, ~45%), data analysis and/or interpretation (299/709, ~42%), and dissemination of the research findings (214/723, ~30%). In 15 of the 18 (83%) reviews, authors indicated that there was a lack of reporting on how and/or when stakeholders were engaged in different stages of the research process.

**Table 3:** Stakeholder engagement in the different phases of the research process based on data from 18 reviews

First author	Planning phase		Conducting phase		Dissemination phase	Number of included studies *(denominator)*	Lack of reporting^**a**^
	*Identifying research questions*	*Developing study protocol*	*Data collection*	*Data analysis and/or interpretation*	*Dissemination of research findings*		
Brear [59]	62%	45%	76%	70%	32%	66	yes
Camden [1]	53%	N.R.	74%	58%	58%	19	yes
Concannan [61]	34%	44%	36%	N.R.	9%	95	yes
Dawson [84]	2%	71%	44%	27%	24%	41	yes
De Las Nueces [62]	63%	74%	63%	58%	47%	19	yes
DiLorito [86]	15%	31%	69%	54%	N.R.	13	no
Forsythe [34]	54%	43%	17%	N.R.	31%	35	yes
Frankena [65]	42%	65%	N.R.	38%	58%	26	yes
Gagliardi [66]	77%	77%	15%	38%	54%	13	yes
Jacquez [88]	77%	84%	84%	54%	52%	56	no
Ragavan [89]	60%	N.R.	N.R.	50%	N.R.	20	yes
Salimi [47]	38%	38%	25%	25%	25%	8	no
Shen [73]	40%	90%	50%	60%	50%	10	yes
Shippee [100]	77%	14%	3%	6%	6%	202	yes
Snijder [49]	32%	42%	55%	N.R.	N.R.	31	yes
Tricco [76]	40%	49%	52%	71%	44%	73	yes
Vaughn [54]	N.R.	80%	76%	75%	N.R.	83	yes
Vishwanathan [92]	47%	47%	83%	65%	68%	60	yes
**Total**	**423/787, ~54%**	**393/831, ~47%**	**374/824, ~45%**	**299/709, ~42%**	**214/723, ~30%**	**870**	**15/18, 83% yes**

*Notes:* The selected reviews (18 out of 86 reviews) were included in this sub-analyses if the review included information on the engagement of stakeholders in at least two different research phases. Full references of the reviews are included in the supplementary file. The percentages in the table indicate the percentage of included studies that reported on the engagement of stakeholders in that specific phase of the research project. The denominator is different for each review as they represent the number of included studies in the concerning review. As we did not check for overlap in the primary studies included in this sub-set of reviews, the total percentages should be interpreted with caution. The total percentages are, therefore, shown as approximates (~). The table on **OSF** includes details on the analysis

*N.R.* Not reported

^a^Yes indicates that the authors of the review mentioned that there was lack of reporting on how and/or when stakeholders were engaged in the different phases of the research process. No indicates that the authors did not include a statement related to reporting on how and/or when stakeholders are engaged in the different phases of the research process

### Terminology, terms and definitions in research partnership literature

As expected, different terms and definitions were used to describe different types of research partnership approaches. In 45 (52%) reviews, authors discussed the challenge of conducting a literature review in the area of research partnerships because of the variation in terms and terminology and/or the lack of reporting on details of the research partnerships processes (OSF – Table III). Table 4 provides a list of key terms used for research partnerships by authors of the included reviews. Nineteen reviews (22%) used a general overarching term to describe the partnership approach such as ‘stakeholder engagement’, ‘community engagement’ or ‘service user engagement’. The overarching terms were terms without a specific focus on research and may therefore also be used in other (review) studies to describe other types of partnerships such as stakeholder engagement in healthcare decisions or policy. We included in this review only reviews that focused on or had some focus on stakeholder engagement in the research process. Other terms to describe research partnerships included CBPR (*n* = 30, 35%), participatory research (PR) (*n* = 8, 9%), patient and public involvement (PPI) (*n* = 7, 8%), PAR (*n* = 5, 6%), action research (*n* = 2, 2%), IKT (*n* = 1) and other terms (*n* = 12, 14%). While authors tended to use one term throughout their review, we found a large variation in the terms used in the title of the primary studies included in the reviews.

**Table 4:** Key terms reported in the included reviews (*n* = 86)

Identified key terms	Number of reviews	Percentage (%) of included reviews (*n* = 86)
Community-based participatory research (CBPR)	30	35%
*Community-based research (n = 1), Photovoice [as CBPR method] (n = 3)*		
Overarching terms	19	22%
*Community engagement (n = 5), community-based organisation engagement (n = 1), consumer engagement (n = 1), community participation (n = 1), patient and public engagement (n = 1), patient involvement (n = 1), patient engagement (n = 2), patient and service user engagement (n = 1), service user engagement (n = 2), stakeholder engagement (n = 3), user engagement (n = 1)*		
Participatory research (PR)	8	9%
*Participatory health research (n = 1), organisational participatory research (n = 1), participatory epidemiology (n = 1), participatory paediatric research (n = 1)*		
Patient and public involvement (PPI)	7	8%
*Peer research (n = 1)*		
Participatory action research (PAR)	5	6%
Action research (AR)	2	2%
Integrated knowledge translation (IKT)	1	1%
Other terms	13	14%
*Inclusive research (n = 2), co-research (n = 2), community-engaged research (n = 2), co-creation (n = 1), community–academic partnerships (n = 1), community–academic research partnerships (n = 1), participatory evaluation (n = 1), research partnerships (n = 1), collaborative research (n = 1), involvement in research (n = 1)*		
Combination of terms^a^	1	1%

*Notes:* The key term is the term used to describe the study aims, Methods and Results sections. This term may differ from the term used in the primary studies included in the review. *Additional information*: Viswanathan et al. [92] published a *CBPR* definition based on 55 articles. Drahota et al. [2] presented a consensus-based term and definition of community–academic partnership

^a^This review [25] focused specifically on a combination of terms for research partnerships

In 25 of the 86 reviews (29%) authors described the research partnership approach without providing a clear definition (OSF – Table III). Of this selection, 13 (52%) reviews focused on CBPR studies. Comparing the definitions between the 4 most frequently used research partnership terms (CBPR, PAR, PR, PPI) showed that definitions of CBPR, PR and PAR varied largely among reviews, while the definitions of PPI were more consistent among the PPI reviews. The majority (71%) of the reviews describing PPI or a related term (peer research), referred to the INVOLVE definition, which is: “*public involvement in research as research being carried out ‘with’ or ‘by’ members of the public rather than ‘to’, ‘about’ or ‘for’ them*” [106]*.* While CBPR, PAR, PR and PPI were used by review authors in different areas and in different population groups (e.g. children/youth, ethnic minority groups), the use of a certain key term seemed to be related to a specific research area. To illustrate, CBPR was the most frequently used term within the population health area, while terms PAR and PPI were most frequently used in the health services and health and social science areas (Table 2).

**Fig. 2:**
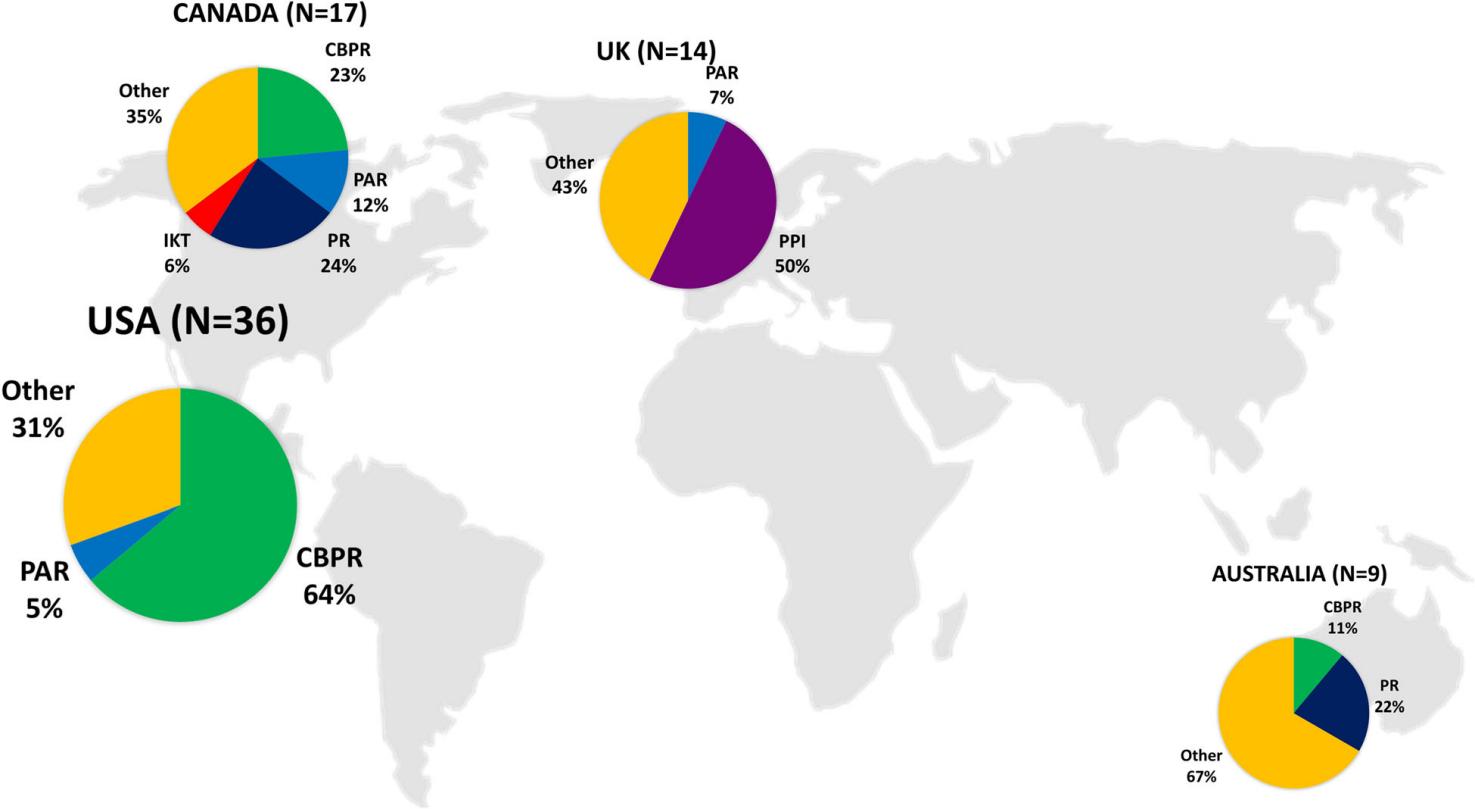
The key terms for research partnerships used by authors from United States, Canada, United Kingdom and Australia. *Notes:* While the term CBPR was most frequently used by authors from the United States, the term PPI was mostly used by authors from the United Kingdom. Similarly, PR is mostly used by review authors from Canada. *N* = 86 reviews. *IKT* integrated knowledge translation, *PAR* participation action research, *CBPR* community-based participatory research, *PPI* patient and public involvement

In contrast to PPI reviews, we found that the definitions using the terms CBPR and PAR tended to highlight the engagement of stakeholders in all phases of the research process. We also found that the choice of a term seemed to be related to the country of the first author of the review. Whereas CBPR was most frequently used by North American researchers, PPI was mainly used by researchers in the United Kingdom. Similarly, PR was most frequently used by researchers from Canada and Australia (Fig. 2). Eight reviews [25, 45, 54, 55, 64, 68, 84, 85] provided an overview of differences and/or similarities of the use of different research partnership terms and/or definitions (Additional file 1: Appendix 6).

#### Terms and terminology key domains

We identified a variety of terms related to principles, strategies, outcomes and impacts of research partnerships (Additional file 1: Appendix 7), indicating that authors used different terms to describe the same concept. To illustrate, 50 out of 80 identified outcome/impact codes (63%) were mentioned by authors of the included reviews using at least two different terms (outcomes, impacts, benefits).

### Principles

After the first analysis round, we extracted 166 principles from the included reviews (OSF – Table IV). The second analysis round resulted in a list of 98 principles and 45 linking values (e.g. trust, respect, credibility, empowerment). We synthesised the 98 principles into 17 overarching principles, and then grouped them into one of the following subcategories (1) relationship between researchers and stakeholders; (2) co-production of knowledge; (3) meaningful stakeholder engagement; (4) capacity-building, support and resources; (5) communication between researchers and stakeholders; and (6) ethical issues of collaborative research activities.

Table 5 describes the final overarching principles. In 7 reviews, no principles were identified (OSF – Table V). The top 3 most frequently identified principles from the included reviews related to the following overarching principles: (1) partners build and maintain relationships based on trust, credibility, respect, dignity and transparency (*n* = 180 of 935 identified principles, 19%); (2) partners co-produce knowledge and meaningfully engage stakeholders at each phase of the research process (*n* = 103, 11%); and (3) partners are flexible and creative in collaborative research activities and tailor the approach (*n* = 72, 8%).

**Table 5:** Overarching principles of research partnerships

No	Principles	Subcategory
1a	Partners build and maintain relationships based on trust, credibility, respect, dignity, and transparency	Relationship between researchers and stakeholders
1b	Partners acknowledge, reward and value the diverse expertise of the partnership and its members	
1c	Partners share in decision-making and leadership of different research activities	
1d	The partnership addresses power dynamics within the team and aim to promote equity, self-determination and/or social justice	
1e	The partnership ensures representation and/or inclusivity and addresses disciplinary and sectoral issues	
2a	Partners co-produce knowledge and meaningfully engage stakeholders at different phases of the research process	Co-production of knowledge
2b	Partners ensure that all members of the partnership have ownership over the data and resulting knowledge products	
2c	Partners strive to balance the need for scientific rigour alongside the practical need for actionable knowledge	
2d	Partners ensure the long-term implementation of the findings in real world settings and systems	
3a	Partners carefully plan and regularly reflect on their strategic approach to collaboration	Meaningful stakeholder engagement
3b	Partners are flexible and creative in the collaborative research activities and tailor the approach	
3c	Researchers and stakeholders benefit from the partnership	
3d	The partnership identifies the stakeholder’s needs and makes sure that the research is relevant for the stakeholders	
4a	Partners build capacity among all members of the partnership	Capacity-building, support and resources
4b	Partners ensure bidirectional exchange of skills, knowledge and capacity between members of the partnership	
5a	The partnership fosters regular, open, clear and honest communication between its members	Communication between researchers and stakeholders
6	Partners address ethical issues related to the collaborative research activities	Ethical issues of collaborative research activities

*Note:* Partners include both researchers and stakeholders. We synthesised the overarching principles from 98 principles. The steps taken to synthesise these overarching principles are described in OSF–Table IV. To help organise these principles, we grouped them into six subcategories. The principles are numbered for feasibility reasons. The order of the principles does not relate to the frequencies

Additional file 1: Appendix 8 describes 13 reviews (13/86; 15%) that explicitly focused on principles of research partnerships [24, 50–52, 55, 58, 68, 72, 83, 89, 96, 101, 103].

### Strategies

After the first analysis round, we extracted 115 strategies from the included reviews (OSF – Table VI). The next round resulted in a list of 111 strategies, which we then synthesised into 11 overarching strategies. To help organise these strategies, we grouped them into one of the following subcategories: (1) relationship between researchers and stakeholders; (2) capacity-building, support and resources; (3) communication between researchers and stakeholders; (4) stakeholder engagement in the planning of the research; (5) stakeholder engagement in conducting the research; and (6) stakeholder engagement in dissemination and application of the research.

While the first three subcategories include strategies that can be used throughout the research process (e.g. relationship, capacity-building, communication), the latter three subcategories include strategies for specific phases of the research project (planning, conducting, dissemination or application). Table 6 describes the final overarching strategies and related subcategories. From almost all of the reviews (*n* = 85, 99%), we extracted at least one strategy (OSF–Table V). The five most frequently identified strategies from the included reviews related to the following overarching strategies: (1) use of a variety of communication strategies (*n* = 183 of 995 identified strategies, 18%); (2) stakeholder engagement in the planning of the research (*n* = 178, 18%); (3) stakeholder engagement in conducting the research (*n* = 159, 16%); (4) stakeholder engagement in dissemination and application of the research (*n* = 155, 16%); and (5) provide opportunities to educate and train all team members (*n* = 87; 9%).

**Table 6:** Overarching strategies of research partnerships

No.	Strategies	Subcategory	
1a	Initiate partnership and identify the team members; the partnership can be initiated by researchers or stakeholders; researchers can use targeted or open strategies to identify the stakeholders	Relationship between researchers and stakeholders	Strategies throughout the research process
1b	Monitor, experiment with and evaluate the collaborative research activities on an ongoing basis		
1c	Work together to develop and define norms, rules and expectations in terms of timelines and tasks; this includes defining the level of stakeholders’ engagement, roles and commitment		
1d	Use a variety of activities to foster collaboration, communication and respect amongst the team members; strategies can include, but are not limited to, creating a common language, negotiating and addressing conflict, tailoring meets to the needs of the team, and providing opportunities to socialise		
2a	Provide opportunities to educate and train all team members; this strategy may include training that supports capacity for collaboration or research methods	Capacity-building, support and resources	
2b	Provide time, resources and funding to support the collaborative research activities; stakeholders may be paid for engagement in the research process		
2c	Provide practical and emotional support to stakeholders to help overcome barriers to engagement		
3a	Use a variety of methods to facilitate communication amongst team members; strategies include, but are not limited to, verbal methods (e.g. structured meetings, brainstorm sessions), written methods (e.g. email discussions, surveys) and visual methods (e.g. photovoice); this communication can be done in-person or via mediated methods (e.g. teleconference, online)	Communication between researchers and stakeholders	
4a	Strategies include, but are not limited to, stakeholder engagement in identifying or refining the ‘research questions’, stakeholder engagement in development the ‘research protocol’, stakeholder engagement developing or refining ‘research instruments’ (e.g. questionnaires, interview guides) and stakeholder engagement in development of participant ‘information material’ (e.g. informed consent)	Stakeholder engagement in the planning of the research	Strategies at specific phases in the research process
5a	Strategies include, but are not limited to, stakeholder engagement in ‘data collection’ (e.g. recruitment of participants, study outcomes, conducting interviews, conducting literature review), stakeholder engagement in data analysis, and interpretation of findings	Stakeholder engagement in conducting the research	
6a	Strategies include, but are not limited to, stakeholder engagement in ‘writing reports or scientific papers’ (e.g. stakeholder is co-author on a scientific paper), stakeholder engagement in ‘presenting findings’ to academic and community audiences, stakeholder engagement in a ‘developing and implementation action plan’ to ensure findings are used, and stakeholders use the findings to create change	Stakeholder engagement in dissemination and application of the research	

*Note:* Partners include both researchers and stakeholders. We synthesised the overarching strategies from 111 strategies extracted from the included reviews. The steps taken to synthesise these overarching strategies are described in OSF-Table V. To help organise these strategies, we grouped them into six subcategories. The strategies are numbered for feasibility reasons. The order of the strategies does not relate to the frequencies

Three Canadian reviews [1, 48, 76] reported the highest number of different strategies (Additional file 1: Appendix 9).

### Outcomes and impacts

One or more outcomes/impacts were extracted from the majority of the reviews (*n* = 74, 86%) (OSF–Table VII). After the analyses, we identified 82 outcomes/impacts from the included reviews. Of these, we classified 56 as beneficial outcomes/impacts (68%) and 26 as challenging or negative outcomes/impacts (32%). We synthesised these outcomes/impacts into 20 overarching outcomes/impacts and clustered them into the following five sub-categories: (1) outcomes and impacts on researchers conducting the partnership research (individual-level); (2) outcomes and impacts on the stakeholder(s) (individual-level); (3) outcomes and impacts on the relationship between researchers and stakeholders (partnership-level); (4) outcomes and impacts on the broader community or society; (5) outcomes and impacts on the research process.

Table 7 outlines the overarching outcomes/impacts including the related subcategories. The top 5 most frequently identified outcomes/impacts from the included reviews were related to the following overarching outcomes/impacts: (1) stakeholders experienced personal benefits from working in a research partnership (*n* = 104 of 675; 15%); (2) partners reported that the research partnership can create high quality research (*n* = 80, 12%); (3) stakeholders experienced increased capacity, knowledge and skills related to research processes (*n* = 74, 11%); (4) partners reported that the research partnership can create increased capacity to conduct and disseminate the research (*n* = 69, 10%); and (5) partners reported that the research partnership can create system changes or action (*n* = 65, 10%).

**Table 7:** Overarching outcomes and impacts

Beneficial outcomes/impacts	Challenging outcomes/impacts	Subcategory
Researchers have experienced increased ‘capacity, knowledge and skills’ related to planning, conducting and disseminating research in partnership with stakeholders; this may include a better understanding of the area under study and/or an increased awareness of community issues		Outcomes and impacts on researchers conducting partnership research (individual level)
Researchers have experienced ‘personal benefits’ from working in a research partnership such as enhanced motivation for the research project and/or lightening of the workload	Researchers have experienced ‘personal challenges’ when working in a research partnership such as uncomfortable feelings when sharing power over the research and/or the additional time and financial burden associated with the research partnership	
Stakeholders have experienced increased ‘capacity, knowledge and skills’ related to research processes; this may include a better understanding of the area under study and/or an increased awareness to the application of the research		Outcomes and impacts on stakeholders involved in research partnerships (individual level)
Stakeholders have experienced a more ‘positive attitude’ towards research and researchers		
Stakeholders have reported better access to information relevant for them such as information on treatments or management of specific diseases or illnesses		
Stakeholders have experienced ‘personal benefits’ from working in a research partnership; examples include, but are not limited to, feeling empowered, feeling valued, increased confidence, increased sense of accomplishment, extended social and support network, and/or increased chances on future employment	Stakeholders have experienced ‘personal challenges’ when working in a research partnership, such as feelings of not being listened to, not being empowered, not being taken seriously, frustrated and/or dissatisfied about the research processes	
	Stakeholders have experienced ‘feeling overburdened’ by tasks and responsibilities	
Partners have reported that the research partnership can ‘have positive outcomes/impacts on the relationship’ between researchers and stakeholders; examples include, but are not limited to, greater partnership synergy, mutual respect, mutual understanding of work style, language, needs and constraints, and/or can create sustainable collaborations	Partners have reported that the research partnership may result in ‘conflicts’ between researchers and stakeholders	Outcomes and impacts on the relationship between researchers and stakeholders (partnership level)
Partners have reported that the research partnership can *‘create system changes or action’* by influencing policy-making, improving community services, improving health-related outcomes for community, and/or creating capacity to sustain the projects		Outcomes and impacts on the community or society
Partners have reported that the research partnership can ‘increase capacity’ in the community by creating better understanding of research in the community and/or increased awareness and knowledge of the study topic		
Partners have reported that the research partnership can increase ‘community empowerment’		
Partners have reported that the research partnership can ‘create community ownership’ of the research		
Partners have reported that the research partnership can increase the ‘acceptability and trust of the research’ in the community		
	Partners have reported that research partnership may create ‘challenging outcomes or impacts on the community’ such as increased time and financial burden on the community organisations, further stigmatisation of the group and/or negative research findings	
Partners have reported that the research partnership can create ‘relevant and useful research findings’		Outcomes and impacts on the research process
Partners have reported that the research partnership can create ‘high quality research’ by generating credible and valid data, developing effective interventions, and/or unearthing new information; the partnership can also general new and other projects		
Partners have reported that the research partnership can create ‘increased capacity’ to conduct and disseminate the research		
	Partners have reported that the research partnership may lead to negative outcomes or impacts, including biased data or tokenism	

*Notes:* Partners include both researchers and stakeholders. As the literature did not differentiate between outcomes and impacts and these terms were used interchangeably throughout the literature, we did not distinguish our results between outcomes and impacts. Challenging outcomes/impacts were also reported in the literature as (potential) negative outcomes/impacts. The order of the outcomes/impacts does not relate to the frequencies

Appendix 10 outlines a description of highlighted reviews specifically focusing on outcomes and/or impacts of research partnerships [4, 28, 60, 66, 80, 81].

#### Potential challenging or negative outcomes/impacts

Although the reviews predominantly reported on the beneficial outcomes/impacts of research partnerships, we also identified potential challenging or negative outcomes/impacts (Table 7). We extracted potential challenging outcomes/impacts at an individual researcher level (e.g. additional time and financial burden, uncomfortable feelings associated with power-sharing), at a partnership level (e.g. conflict between researchers and stakeholders) as well as at the research project level (e.g. biased data). We extracted potential challenging or negative outcomes/impacts at the individual stakeholder level (e.g. feelings of tokenism, disempowerment, overburdened) in reviews related to special populations such as children and youth [87, 104], parents [73], people with intellectual disabilities [65, 86, 90], ethnic minority groups and patient groups [1, 80]. We did not identify potential negative outcomes/impacts at the individual stakeholder level in reviews (*n* = 3) on partnerships with organisations or policy-makers, decision-makers or managers [60, 66, 105].

## Discussion

This review of reviews provides a guide through the diverse literature on research partnerships in different research areas and with different stakeholder groups. We identified an extensive set of research partnership principles, strategies, outcomes and impacts from the included reviews.

### Principles and strategies

We synthesised information on principles and strategies from a variety of research partnership approaches into 17 overarching principles and 11 overarching strategies. As these overarching principles and strategies are based on reviews (instead of primary studies), we synthesised them in a broad and general way. As such, the overarching principles and strategies may not directly apply to all research partnerships as these are context dependent. Three key research partnership characteristics may guide the contextualisation of these principles and strategies. The first characteristic is ‘the stakeholder group’. To illustrate, we saw that different principles and strategies are used when working with different groups of stakeholders (e.g. people with lived experience with a health condition versus policy-makers versus community organisations). The second characteristic is ‘the level of engagement’. Principles and strategies should align with the level of stakeholder engagement, which may be determined by using the five engagement categories of the widely used IAP2 Spectrum of Public Participation (e.g. inform, consult, involve, collaborate, empower) [107] or using other engagement frameworks (e.g. [3, 108, 109]). The third characteristic is ‘the research phase’. While some principles and strategies may be applicable throughout the research phase, others may be more important or applicable for specific phases of the research project [76].

To gain a better understanding of which principles and strategies are successful in which contexts and under which circumstances, more detailed reporting and consistent use of related terms across the research partnership literature at both the individual study- and review-level is required. Our review might be used as a first step in developing a classification system of principles and strategies for research partnership approaches to improve the consistency of reporting (e.g. similar to Michie’s behaviour change technique taxonomy [110] and Hoffman et al. [111] reporting for interventions). However, it is likely that further examination of primary research studies are needed before such classification systems can be established.

### Outcomes and impacts

Our results show that outcomes and impacts are not well-differentiated and that these terms are used interchangeably throughout the reviews examined. This finding may suggest that authors are not aware of potential conceptual differences between outcomes and impacts. To gain a better understanding about specific outcomes and specific impacts of research partnerships, further research is needed in which data related to these key domains are extracted from primary studies (as opposed to reviews) using specific definitions [11].

While the literature predominantly highlighted the positive outcomes and impacts of research partnership approaches, we found that reviews also reported on potential challenging or negative outcomes/impacts (Table 7). The question then arises of whether such potential negative outcomes/impacts may be a result of poor relationships between researchers and stakeholders (i.e. failed partnerships), poor co-production processes, a combination of both and/or other partnership influences. In our review of reviews, we were unable to answer this question, because of the high-level focus of this review. A different research design, such as a realist review [112, 113] or interviews, would be more appropriate to answer these type of research questions. While more in-depth studies are needed to explore how, when and why research partnership approaches are perceived to be beneficial or not, a recent commentary paper by Oliver et al. [114] provides initial guidance.

Studies evaluating research partnership approaches are scarce and mainly focus on perceived and self-reported outcomes/impacts. More in-depth, prospective multi-case studies are needed to advance the science of research partnerships (e.g. [7, 15]). To provide further guidance on ‘how to study a research partnership’, our subsequent scoping reviews will identify tools, methods and methodologies to evaluate research partnership approaches [11]. These insights should help researchers to better monitor, evaluate and report their partnership approaches as well as contribute to more high-quality data on outcomes and impacts of research partnership approaches at both study and review level.

### Limitations and strengths

The first limitation relates to the qualitative nature of our data. We extracted qualitative data from the included reviews without verifying the data with the primary studies. This may have resulted in inaccurate and/or biased findings. Moreover, the interpretations of the reviews may be flawed due to a lack of details and/or differences in research partnership (domain) terms, terminology and definitions used in the primary studies and/or reviews. For this reason, we were reluctant to report on the number of times (frequencies) that a specific finding (principles, strategies, outcome, impact) had been reported in the reviews. We aimed to address this limitation by focusing this review on high-level findings on research partnerships domains without providing details on what worked best under which circumstances.

The second limitation relates to our eligibility criteria. We excluded articles that did not use a systematic search (e.g. [115, 116]) and/or used other types of partnerships (e.g. public–private partnerships, partnership in healthcare, partnership in education). We also excluded reviews that did not include a specific aim or sub-aim related to research partnerships. By excluding these and other reviews, we may have missed relevant information related to key domains of research partnerships.

The third limitation relates to our search strategy. In line with the focus on our review, we used a very high-level search strategy to capture relevant reviews. We omitted some relevant concepts in our search strategy, including terms related to ‘stakeholder engagement’, ‘patient and public involvement’, and ‘implementation’. By doing so, we realised and accepted that we might have missed potentially relevant reviews in our first step of synthesising the research partnership literature, in particular those that were reviewing particular partnership methodologies in the research process. For our next steps, which includes conducting three scoping reviews, we have developed a series of more comprehensive search strategies informed by our findings from this review of reviews. The steps taken to develop the search strategy capturing the concept of ‘research partnership’ are described elsewhere [117]. We would encourage individuals interested in doing a review related to research partnership to use and build upon our comprehensive search strategy.

The fourth limitation relates to our extraction process. As the extraction process was time consuming because of the qualitative nature of the data, we did not extract the data in duplicate. However, the data extraction process began only after consensus-driven and reliable coding methods were established. The synthesised processes of our overarching principles, strategies and outcomes/impacts were conducted by project leads (FH, HG) and critically reviewed by other members of the research team. To improve transparency, the steps taken to synthesise our overarching findings are available on OSF.

The final limitation relates to our quality assessment process. We used a quality assessment tool (R-AMSTAR) that was primarily developed to assess the quality of systematic reviews in clinical settings. As different review types have different goals (e.g. summarising existing knowledge versus data aggregation) with different corresponding methods, our quality assessment tool may not be preferred to assess the quality of other types of reviews (e.g. scoping reviews, narrative reviews, realist reviews) [118]. Because of the limitations of our quality assessment approach, we did not use these results to synthesise our overarching findings. Alternatively, we included quality assessment scores and corresponding percentile grades to orient the reader.

This review has several strengths. First, this is the first review of reviews focusing on different types of research partnerships in a variety of research areas and thus may serve as a guide through the research partnership literature.

Second, we used a collaborative research approach (Coordinated Multicenter Team) [11] and we engaged stakeholders (steering committee) (Additional file 1: Appendix 3) in different phases of the project (Additional file 1: Appendix 4), strengthening our procedures and findings.

Third, our processes are clearly documented and presented transparently. Details related to our search strategy as well as different steps in the analysis process are available on OSF. By doing so, other researchers who are planning to synthesise the literature on research partnerships can build upon our methods and findings.

Finally, this review is part of a collaborative review approach and has both theoretical and practical contributions. To the research partnership literature, our findings provide new insights on key domains (principles, strategies, outcomes, impacts) of research partnership approaches across a variety of research areas. We hope that our collaborative review efforts will contribute to better and more consistent reporting of the research partnership literature and ultimately advance the science of research partnerships. Moreover, the findings from this review may help researchers and stakeholders from different research areas to plan, conduct and/or disseminate research in partnership (Table 8). Ultimately, we hope that our collaborative review efforts will contribute to improving the quality and conduct of research partnerships in many different areas across the world.

**Table 8:** Initial guidance for the use of our findings by research partnerships

Summarising steps of research partnership processes		Additional information
**Step 1**	**Build and maintain relationships** between academic researchers and stakeholders; the relationship may be built upon values important for all partnership members such as trust, respect, transparency, credibility	Table 7 provides strategies related to building and maintaining relationships; OSF-Table IV provides a list of identified principles and values
**Step 2**	**Determine the level of stakeholder engagement** (e.g. inform, consult, involve, collaborate, empower) for each phase in the research process (planning phase, conducting phase, disseminating phase)	Table 7 provides strategies for different phases in the research process and OSF-Table IV provides a list of related principles; two reviews [25, 76] included other examples of detailed frameworks of the use of collaborative research activities in different phases of the research process
**Step 3**	Contextualisation: **Select and/or adapt principles and strategies relevant for your research partnership in your research area**; principles and strategies need to align with the desirable level of stakeholder engagement and need to align with the needs and preferences of all members of the partnership; principles and strategies may differ between different phases of the research process	Table 2 provides an overview of the research areas and population of the included reviews; in addition to the related reviews, we recommend exploring individual studies that used or evaluated research partnership approaches specifically related to your research area and/or population; an example of the contextualisation for spinal cord injury research partnerships is described elsewhere [OSF]
**Step 4**	**Communicate, monitor, and report the principles, strategies, outcomes and impacts** of the research partnership; this information will provide the opportunities to learn from your own as well as from others successes and challenges related to collaborative research activities, and may contribute to advancing the science of research partnerships	To support consistent reporting, Additional file 1: Appendix 1 provides our consensus-based guiding framework including definitions of the key domains of research partnerships

*Notes:* We identified these four steps based on our own experiences from reviewing the research partnership literature as well as our own experiences with conducting and disseminating research in partnerships; these steps may help readers to tailor our overarching findings of research partnerships processes to their local context. Our next umbrella review will include more specific recommendations

## Future directions

Due to the variation in terms, terminology and definitions in combination with the lack of reporting on details related to the research partnership approaches, we were unable to create a systematic overview of the differences and similarities of the different research approaches. Further research using different research designs is needed to identify and understand the similarities and differences between different partnership approaches (e.g. [119]). We found that the use of partnership terms seemed to be associated with the research area (Table 2) and country of the first author (Fig. 2). To ensure the consistency of terms and definitions, researchers from different areas and from different countries should work together to build consensus on common research partnership terms, terminology and definitions.

As funding agencies are increasingly promoting the use of research partnership approaches, additional guidance may be needed to support researchers and stakeholders in establishing and conducting partnered research. Therefore, future research should focus on the development of evidence-based support services, including tools and resources for research partnerships related to the key domains (principles, strategies, outcomes, impacts).

Our overarching findings are extracted from the diverse research partnership literature and are not tailored to specific groups of stakeholders or specific research areas. Additional efforts, including the contextualisation of research partnership processes, may be needed. Based on our own experiences, we identified four steps that may be used to tailor our overarching findings of research partnership processes to local settings (Table 8).

## Conclusions

This review of reviews is the first to present the overarching principles, strategies, outcomes and impacts of research partnerships. This review is unique in scope as we synthesised the literature in different research areas that included different stakeholder groups. By doing so, this review begins to map the diverse research partnership literature. The overarching principles, strategies and outcomes/impacts can be a first step towards creating a classification system of these domains, which may be used to guide researchers and partnerships, improve the consistent reporting of these domains in the literature, and will ultimately help to advance the science and practice of research partnerships.

## Supplementary information

**Additional file 1.**

## Data Availability

Search strategies for individual databases, references of included and excluded papers, and details related to data analyses are available through Open Science Framework.

## Abbreviations

IKT: Integrated knowledge translation
PAR: Participatory action research
PR: Participatory research
CBPR: Community-based participatory research
PPI: Patient and public involvement
OSF: Open Science Framework
SCI: Spinal cord injury
