# Supplementary material for: A review of reviews on principles, strategies, outcomes and impacts of research partnerships approaches: a first step in synthesising the research partnership literature
Source: Health Res Policy Syst. 2020 May 25;18:51. doi: 10.1186/s12961-020-0544-9 (PMC7249434; doi:10.1186/s12961-020-0544-9)
Supplement: Supplementary file 1 — Additional file 1. [file 12961_2020_544_MOESM1_ESM.zip › 12961_2020_544_MOESM1_ESM/Supplementary files Review of Reviews_Total.pdf]

Supplementary files - Principles, strategies, outcomes, and impacts of research partnerships approaches: a review of reviews

- **Appendix 1**: Guiding framework and definitions
- **Appendix 2:** PRISMA-P guidelines
- **Appendix 3:** Members of the SCI Guiding Principles Consensus Panel (Steering committee)
- **Appendix 4:** Stakeholder engagement
- **Appendix 5:** Reference list of included reviews (n=86).
- **Appendix 6.** Terms and definitions: Reviews that reported on differences and similarities of different research partnership terms and definitions.
- **Appendix 7:** List of identified terms to describe key domains
- **Appendix 8**: Principles: description of reviews that specifically focused on principles of research partnerships
- **Appendix 9:** Strategies: description of the reviews including the highest number of different strategies.
- **Appendix 10**: Outcomes and Impacts: Description of highlighted reviews on outcomes and/or impacts of research partnerships.

Additional files supporting our findings are published on Open Science Framework (OSF)

- Hoekstra, F., Mrklas, K. J., Nguyen, T., Vis-Dunbar, M., Sibley, K., & Gainforth, H. L. (2020, March 15). Review of Reviews. https://doi.org/10.17605/OSF.IO/WBFDE

List of Tables on Open Science Framework:

- Table I – Search strategy review of reviews
- Table II – Engagement of stakeholders in research process
- Table III – Review and partnership characteristics
- Table IV – Principles final
- Table V – Number of principles strategies outcomes impacts
- Table VI – Strategies final

- Table VII – Outcomes and impacts final
- Table VIII – Quality assessment

Appendix 1: Guiding framework and definitions

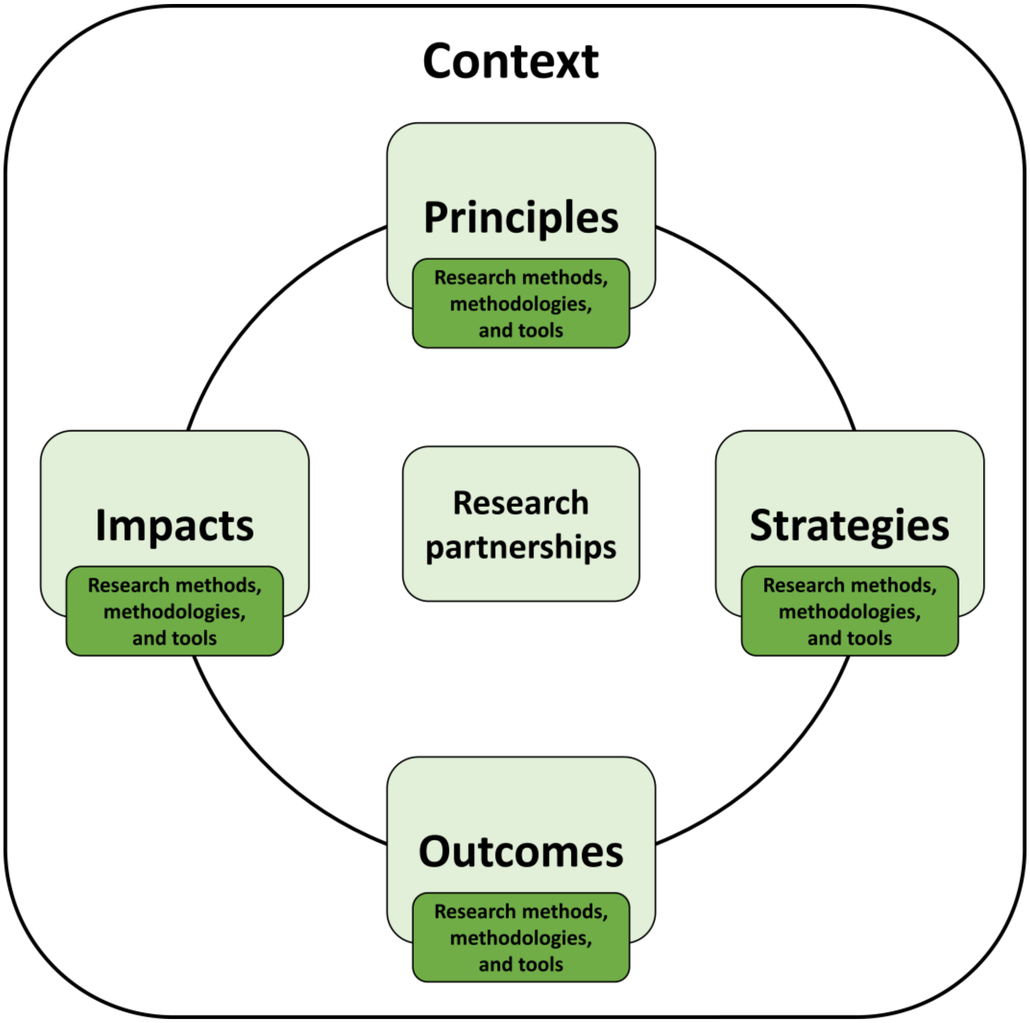

Figure A1: The Guiding Framework

Figure A1 presents the Guiding Framework that is developed by the Coordinated Multicenter Team. The Framework includes the four key domains (principles, strategies, outcomes, impacts) of research partnership approaches.

As described in our protocol paper [1], the following operational terms and definition are used in the review of reviews:

- Research partnerships: "individuals, groups or organizations engaged in collaborative research activity involving at least one researcher (e.g., individual

affiliated with an academic institution), and any stakeholder actively engaged in any part of the research process (e.g., decision or policy maker, health care administrator or leader, community agency, charities, network, patients etc.)". Examples of research partnership approaches include, but are not limited to, IKT, participatory research, and participatory action research.

- Principles: "fundamental norms, rules, of beliefs that represent what is desirable and positive for a person, group, organization, or community, and help it in determining the rightfulness or wrongfulness of its actions. Principles are more basic than policy and objectives, and are meant to govern both".
- Strategies: "Observable actions designed to achieve an outcome".
- Outcomes: "A planned, a priori assessment described in the study methods that is used to determine a change in status as a result of interventions, can be measured or assessed as a component of the study and is not something of futuristic benefit".
- Impacts: "Identifiable benefit to, or positive influence on, the economy, society, public services, health, the environment, quality of life, or academia".[1]
- Context: defined as "the physical, organizational, institutional and legislative structures that enable and constrain, and resource and realize, people and procedures"

References:

- Hoekstra F, Mrklas KJ, Sibley KM, Nguyen T, Vis-Dunbar M, Neilson CJ, Crockett LK, Gainforth HL, Graham ID: A review protocol on research partnerships: a Coordinated Multicenter Team approach. *SYSTEMATIC REVIEWS* 2018, 7(1):217. doi: 10.1186/s13643-018-0879-2

[1] As the literature did not differentiate between outcomes and impacts in a consistent way and we did not check our extracted data with the primary studies, we decided to extract outcome and impact data together using both definitions. In addition to that, we noticed that the literature also reported on potential challenging or negative impacts. As we wanted to provide an overview of the literature, we decided to extract these potential negative or challenging impacts as well.

Appendix 2: PRISMA guidelines

The PRISMA guideline checklist is attached as a separated file.

Appendix 3: Members of the SCI Guiding Principles Consensus Panel (Steering committee).

Panel member	Organization(s)	Roles
Kim Anderson	NASCIC	Researcher + person with lived experience of SCI
Hugh Anton	ICORD, UBC Medicine	Clinician + researcher
Peter Athanasopoulos	SCI Ontario, SCI Solutions Alliance	Leadership role in community organization + person with lived experience of SCI
John Chernesky	RHI, NASCIC	Leadership role in funding organization + person with lived experience of SCI
Susan Forwell	ICORD, UBC Occupational Therapy	Clinician + researcher
Jocelyn Maffin	SCI BC	Representative from community organisation + person with lived experience of SCI
Kathleen Martin Ginis	ICORD, UBC	Researcher
Christopher B McBride	SCI Canada, SCI BC	Executive director of a community organization
W. Ben Mortenson	ICORD, UBC	Clinician + researcher
Rhonda Willms	ICORD, GF Strong, UBC Medicine	Clinician + researcher

Notes: The SCI Guiding Principles Consensus Panel was established in 2017. NASICIC = North American SCI Consumer Consortium; ICORD = International Collaboration on Repair Discoveries; UBC = University of British Columbia; SCI = Spinal Cord Injury; RHI = Rick Hanson Institute;

Appendix 4: Stakeholder engagement in the design and conduct of the review of reviews.

The research question related to developing guiding principles and strategies for Integrated Knowledge Translation (IKT) derived from members of the Spinal Cord Injury (SCI) Guiding Principles Consensus Panel. To gain a further understanding of the use of research partnership approaches and to align with the focus of our Coordinated Multicenter Team, this review of reviews focused on not only principles and strategies, but also outcomes and impacts. To prevent overburden of our partners, we only engaged stakeholders in the parts related to the 'principles' and 'strategies'. We did not discuss the results related to outcomes and impacts with our stakeholders (SCI consensus panel). However, panel members had the opportunity to provide feedback on the manuscript, prior to submission.

We acknowledged that in the context of this broad review of reviews, the scope of our panel members was specific to SCI research. It is possible that results would have been interpreted and synthesized in a different way if we would have involved a more diverse group of stakeholders with other backgrounds (beyond SCI), and involved them in the interpretation of all results, including the outcomes/impacts. To improve transparency of our collaborative research activities, we provided details on panel members' names and roles (**Appendix 3**) as well as the how they were engaged in this review of reviews (**Table A4**). The table below provides an overview of research activities, associated dates, topics discussed, stakeholders' concerns and suggestions, and our responses.

Table A4: Stakeholder engagement in the review process

Research activity	Date	Topics discussed	Concerns and suggestions from panel	Our responses
Conceptual design and formulation of the research questions	13 September 2017	One-day meeting to establish the consensus panel. During this meeting, the need for a literature review focusing on principles and strategies of research partnership was discussed and outlined as a priority for the panel.	-	-
	4 December 2017	A conference call to discuss the establishment of a Coordinated Multicenter Team Approach to conduct the proposed reviews.	Panel mentioned that they were concerned about the timeline, as they did not ask for a review of reviews. The panel approved the approach as long as it would not result in a heavy delay of the development of the IKT SCI guiding principles.	We discussed the expected timelines and highlighted that the review of reviews would increase the rigor of development of the IKT SCI guiding principles.
	29 January 2018 and 22 March 2018	Email contact in which we circulated our protocol paper of the Coordinator Multicenter Team approach.	Panel suggested to list the members of the steering committee including their roles and to distinguish between 'patients' and 'people with lived experience'.	The protocol paper includes the participating organizations, the current review includes panel members names, organizations, and roles (see **Appendix 3**).
Before starting data extraction	May 8, 2018	Conference call to discuss and approve plans for data extraction.	Panel members suggested extracting information about motivators of research partnerships.	As this review of reviews provides overarching findings of key domains of research partnership, we did not extract motivators of partnerships. However, we will extract this in our scoping review.
Data analysis, interpretation, and dissemination of results.	March 4, 2019	One conference call meeting to discuss the findings related to the overarching principles and strategies. Panel members provided feedback on the findings.	Panel members suggested to restructure the overarching principles and strategies – as the presented 'themes' did not align with the presented principles/strategies. Panel members also suggested including	We re-structured our overarching principles/strategies. We removed the 'themes' and changed them to 'processes' (see **OSF**). We did not include examples of principles/strategies in this review because of the lack of detail presented in the reviews and the diversity

			examples of overarching principles/strategies.	of the literature. We will include examples of the principles/strategies in our next scoping review related to disability research partnerships.
	August 28, 2019	Email contact in which we circulated the manuscript for feedback and final approval.	Two panel members provided feedback on the manuscript (minor revisions)	

IKT = Integrated Knowledge Translation; SCI = Spinal Cord Injury

Appendix 5: Reference list of included reviews (n=86).

As described in the manuscript, this review of reviews did not aim to provide a comprehensive overview of the research partnership literature. As such, it is possible that we might have missed potentially relevant reviews in our first step of synthesizing the research partnership literature.

Adams K, Faulkhead S: This is not a guide to indigenous research partnerships But it could help. Information communication & Society 2012, 15:1016-1036.

Adebayo OW, Salerno JP, Francillon V, Williams JR: A systematic review of components of community-based organisation engagement. Health & Social Care in the Community 2018.

Andrews JO, Newman SD, Heath J, Williams LB, Tingen MS: Community-Based Participatory Research and Smoking Cessation Interventions: A Review of the Evidence. Nursing Clinics of North America 2012, 47:81-96.

Bach M, Jordan S, Hartung S, Santos-Hoevener C, Wright MT: Participatory epidemiology: the contribution of participatory research to epidemiology. Emerging themes in epidemiology 2017: 14:2

Backhouse T, Kenkmann A, Lane K, Penhale B, Poland F, Killett A: Older care-home residents as collaborators or advisors in research: a systematic review. Age and aging 2016, 45:337-345.

Bailey S, Boddy K, Briscoe S, Morris C: Involving disabled children and young people as partners in research: a systematic review. Child: Care, Health and Development 2015, 41:505-514.

Baines RL, de Bere SR: Optimizing patient and public involvement (PPI): Identifying its “essential” and “desirable” principles using a systematic review and modified Delphi methodology. Health expectations 2018, 21:327-335.

Blair T, Minkler M: Participatory Action Research With Older Adults: Key Principles in Practice. Gerontologist 2009, 49:651-662.

Brear M, Hammarberg K, Fisher J: Community participation in research from resource-constrained countries: a scoping review. Health Promotion International 2018, 18:18.

Brett J, Staniszewska S, Mockford C, Herron-Marx S, Hughes J, Tysall C, Suleman R: A Systematic Review of the Impact of Patient and Public Involvement on Service Users, Researchers and Communities. Patient-patient centered outcomes research 2014, 7:387-395.

Brett J, Staniszewska S, Mockford C, Herron-Marx S, Hughes J, Tysall C, Suleman R: Mapping the impact of patient and public involvement on health and social care research: a systematic review. Health expectations 2014, 17:637-650.

Brizay U, Golob L, Globerman J, Gogolishvili D, Bird M, Rios-Ellis B, Rourke SB, Heidari S: Community-academic partnerships in HIV-related research: A systematic literature review of theory and practice. Journal of the International AIDS Society 2015, 18.

Brown Speights JS, Nowakowski ACH, De Leon J, Mitchell MM, Simpson I: Engaging African American women in research: an approach to eliminate health disparities in the African American community. Family Practice 2017, 34:322-329.

Bush PL, Pluye P, Loignon C, Granikov V, Wright MT, Pelletier J-F, Bartlett-Esquilant G, Macaulay AC, Haggerty J, Parry S et al: Organizational participatory research: a systematic mixed studies review exposing its extra benefits and the key factors associated with them. Implementation science 2017, 12.

Camden C, Shikako-Thomas K, Nguyen T, Graham E, Thomas A, Sprung J, Morris C, Russell DJ: Engaging stakeholders in rehabilitation research: a scoping review of strategies used in partnerships and evaluation of impacts. Disability and rehabilitation 2015, 37:1390-1400.

Carter MW, Tregear ML, Lachance CR: Community Engagement in Family Planning in the U.S.: A Systematic Review. American journal of preventive medicine 2015, 49:S116-123.

Catalani C, Minkler M: Photovoice: A Review of the Literature in Health and Public Health. Health education & behavior 2010, 37:424-451.

Chen PG, Diaz N, Lucas G, Rosenthal MS: Dissemination of Results in Community-Based Participatory Research. American journal of preventive medicine 2010, 39:372-378.

Commodore A, Wilson S, Muhammad O, Svendsen E, Pearce J: Community-based participatory research for the study of air pollution: a review of motivations, approaches, and outcomes. Environmental Monitoring and Assessment 2017, 189.

Concannon TW, Fuster M, Saunders T, Patel K, Wong JB, Leslie LK, Lau J: A Systematic Review of Stakeholder Engagement in Comparative Effectiveness and Patient-Centered Outcomes Research. Journal of general internal medicine 2014, 29:1692-1701.

Cook WK: Integrating research and action: a systematic review of community-based participatory research to address health disparities in environmental and occupational health in the USA. Journal of epidemiology and community health 2008, 62:668-676.

Coons KD, Watson SL: Conducting research with individuals who have intellectual disabilities: Ethical and practical implications for qualitative research. Journal on Developmental Disabilities 2013, 19:14-24.

Coughlin SS, Smith SA: Community-Based Participatory Research to Promote Healthy Diet and Nutrition and Prevent and Control Obesity Among African-Americans: a Literature Review. Journal of racial and ethnic health disparities 2017, 4:259-268.

Crabtree CA: A community-based participatory research approach to assist vulnerable communities in developing a disaster preparedness plan to lessen the impact of natural disasters. 2014, 74.

Cyril S, Smith BJ, Possamai-Inesedy A, Renzaho AMN: Exploring the role of community engagement in improving the health of disadvantaged populations: a systematic review. Global health action 2015, 8:1-12.

Dawson S, Campbell SM, Giles SJ, Morris RL, Cheraghi-Sohi S: Black and minority ethnic group involvement in health and social care research: A systematic review. Health expectations 2018, 21:3-22.

De Las Nueces D, Hacker K, DiGirolamo A, Hicks LS: A Systematic Review of Community-Based Participatory Research to Enhance Clinical Trials in Racial and Ethnic Minority Groups. Health services research 2012, 47:1363-1386.

Dempsey JS: Using photovoice to identify perceived risk and protective factors for rural adolescent depression. 2015, 75.

Di Lorito C, Birt L, Poland F, Csipke E, Gove D, Diaz-Ponce A, Orrell M: A synthesis of the evidence on peer research with potentially vulnerable adults: how this relates to dementia. International Journal of Geriatric Psychiatry 2017, 32(1):58-67.

Di Lorito C, Bosco A, Birt L, Hassiotis A: Co-research with adults with intellectual disability: A systematic review. Journal of Applied Research in Intellectual Disabilities 2018, 31(5):669-686.

Domecq JP, Prutsky G, Elraiyah T, Wang Z, Nabhan M, Shippee N, Brito JP, Boehmer K, Hasan R, Firwana B et al: Patient engagement in research: a systematic review. BMC Health Services Research 2014, 14:89.

Drahota A, Meza RD, Brikho B, Naaf M, Estabillo JA, Gomez ED, Vejnoska SF, Dufek S, Stahmer AC, Aarons GA: Community-Academic Partnerships: A Systematic Review of the State of the Literature and Recommendations for Future Research. The Milbank quarterly 2016, 94:163-214.

Ehde DM, Wegener ST, Williams RM, Ephraim PL, Stevenson JE, Isenberg PJ, MacKenzie EJ: Developing, Testing, and Sustaining Rehabilitation Interventions Via Participatory Action Research. Archives of Physical Medicine and Rehabilitation 2013, 94:S30-S42.

Eyles H, Jull A, Dobson R, Firestone R, Whittaker R, Te Morenga L, Goodwin D, Mhurchu CN: Co-design of mHealth Delivered Interventions: A Systematic Review to Assess Key Methods and Processes. Current Nutrition Reports 2016, 5(3):160-167.

Forsythe LP, Szydlowski V, Murad MH, Ip S, Wang Z, Elraiyah TA, Fleurence R, Hickam DH: A systematic review of approaches for engaging patients for research on rare diseases. J Gen Intern Med 2014, 29 Suppl 3:S788-800.

Fouché CB, Chubb LA: Action Researchers Encountering Ethical Review: A Literature Synthesis on Challenges and Strategies. Educational Action Research 2017, 25(1):23-34.

Frankena TK, Naaldenberg J, Cardol M, Linehan C, Lantman-de Valk HvS: Active involvement of people with intellectual disabilities in health research - A structured literature review. Research in developmental disabilities 2015, 45-46:271-283.

Gagliardi AR, Berta W, Kothari A, Boyko J, Urquhart R: Integrated knowledge translation (IKT) in health care: a scoping review. Implementation Science 2016, 11.

Ganann R: Opportunities and challenges associated with engaging immigrant women in participatory action research. Journal of immigrant and minority health / Center for Minority Public Health 2013, 15(2):341-349.

Greenhalgh T, Jackson C, Shaw S, Janamian T: Achieving Research Impact Through Co-creation in Community-Based Health Services: Literature Review and Case Study. Milbank quarterly 2016, 94:392-429.

Haijes HA, van Thiel GJMW: Participatory methods in pediatric participatory research: a systematic review. PEDIATRIC RESEARCH 2016, 79:676-683.

Harrop JP, Nelson DE, Kuratani DG, Mullen PD, Paskett ED: Translating Cancer Prevention and Control Research into the Community Setting: Workforce Implications. Journal of cancer education 2012, 27:157-164.

Hergenrather KC, Rhodes SD, Cowan CA, Bardhoshi G, Pula S: Photovoice as community-based participatory research: a qualitative review. American journal of health behavior 2009, 33(6):686-698.

Hubbard G, Kidd L, Donaghy E: Involving people affected by cancer in research: a review of literature. European journal of cancer care 2008, 17:233-244.

Jacquez F, Vaughn LM, Wagner E: Youth as Partners, Participants or Passive Recipients: A Review of Children and Adolescents in Community-Based Participatory Research (CBPR). American journal of community psychology 2013, 51:176-189.

Jagosh J, Macaulay AC, Pluye P, Salsberg J, Bush PL, Henderson J, Sirett E, Wong G, Cargo M, Herbert CP et al: Uncovering the Benefits of Participatory Research: Implications of a Realist Review for Health Research and Practice. Milbank quarterly 2012, 90:311-346.

Jivraj J, Sacrey L-A, Newton A, Nicholas D, Zwaigenbaum L: Assessing the influence of researcher-partner involvement on the process and outcomes of participatory research in autism spectrum disorder and neurodevelopmental disorders: A scoping review. Autism 2014, 18:782-793.

Joss N, Cooklin A, Oldenburg B: A scoping review of end user involvement in disability research. Disabil Health J 2016, 9(2):189-196.

Krishnaswami J, Martinson M, Wakimoto P, Anglemeyer A: Community-Engaged Interventions on Diet, Activity, and Weight Outcomes in U.S. Schools A Systematic Review. American journal of preventive medicine 2012, 43:81-91.

Kwan C, Walsh CA: Ethical Issues in Conducting Community-Based Participatory Research: A Narrative Review of the Literature. The Qualitative Report 2018, 23(2):369-386.

Lesser J, Oscós-Sánchez MA: Community-academic research partnerships with vulnerable populations. Annual review of nursing research 2007, 25:317-337.

Manafo E, Petermann L, Vandall-Walker V, Mason-Lai P: Patient and public engagement in priority setting: A systematic rapid review of the literature. Plos one 2018, 13.

McVicar A, Munn-Giddings C, Seebohm P: Workplace stress interventions using participatory action research designs. International Journal of Workplace Health Management 2013, 6:18-37.

Mikesell L, Bromley E, Khodyakov D: Ethical Community-Engaged Research: A Literature Review. American journal of public health 2013, 103:E7-E14.

Miller J, Knott VE, Wilson C, Roder D: A review of community engagement in cancer control studies among Indigenous people of Australia, New Zealand, Canada and the USA. European Journal of Cancer Care 2012, 21(3):283-295.

Nilsen ES, Myrhaug HT, Johansen M, Oliver S, Oxman AD: Methods of consumer involvement in developing healthcare policy and research, clinical practice guidelines and patient information material. Cochrane Database of Systematic Reviews 2006:CD004563.

Nitsch M, Waldherr K, Denk E, Griebler U, Marent B, Forster R: Participation by different stakeholders in participatory evaluation of health promotion: A literature review. Evaluation and program planning 2013, 40:42-54.

Noh H, de Sayu RP, Anderson KG, Ford CD: Community-Based Participatory Research on Issues Around Palliative and End-of-Life Care: Literature Review. Journal of hospice & palliative nursing 2016, 18:249-255.

Nwanyanwu K, Grossetta Nardini HK, Shaughness G, Nunez-Smith M, Newman-Casey P: Systematic review of community-engaged research in ophthalmology. Expert Review of Ophthalmology 2017, 12:233-241.

Orlowski SK, Lawn S, Venning A, Winsall M, Jones GM, Wyld K, Damarell RA, Antezana G, Schrader G, Smith D et al: Participatory Research as One Piece of the Puzzle: A Systematic Review of Consumer Involvement in Design of Technology-Based Youth Mental Health and Well-Being Interventions. JMIR Human Factors 2015, 2:e12.

Portillo CJ, Waters C: Community partnerships: the cornerstone of community health research. Annual review of nursing research 2004, 22:315-329.

Ragavan MI, Thomas K, Medzhitova J, Brewer N, Goodman LA, Bair-Merritt M: A systematic review of community-based research interventions for domestic violence survivors. Psychology of Violence 2019 9(2), 139–155.

Salimi Y, Shahandeh K, Malekafzali H, Loori N, Kheiltash A, Jamshidi E, Frouzan AS, Majdzadeh R: Is Community-based Participatory Research (CBPR) Useful? A Systematic Review on Papers in a Decade. International journal of preventive medicine 2012, 3:386-393.

Salsberg J, Parry D, Pluye P, Macridis S, Herbert CP, Macaulay AC: Successful strategies to engage research partners for translating evidence into action in community health: A critical review. Journal of Environmental and Public Health 2015, 2015.

Shen S, Doyle-Thomas KAR, Beesley L, Karmali A, Williams L, Tanel N, McPherson AC: How and why should we engage parents as co-researchers in health research? A scoping review of current practices. Health expectations 2017, 20:543-554.

Shippee ND, Garces JPD, Lopez GJP, Wang Z, Elraiyah TA, Nabhan M, Brito JP, Boehmer K, Hasan R, Firwana B et al: Patient and service user engagement in research: a systematic review and synthesized framework. Health expectations 2015, 18:1151-1166.

Smith E, Ross F, Donovan S, Manthorpe J, Brearley S, Sitzia J, Beresford P: Service user involvement in nursing, midwifery and health visiting research: A review of evidence and practice. International journal of nursing studies 2008, 45:298-315.

Snijder M, Shakeshaft A, Wagemakers A, Stephens A, Calabria B: A systematic review of studies evaluating Australian indigenous community development projects: the extent of community participation, their methodological quality and their outcomes. bmc public health 2015, 15.

Soh KL, Davidson PM, Leslie G, Rahman ABA: Action research studies in the intensive care setting: A systematic review. International Journal of Nursing Studies 2011, 48:258-268.

Souleymanov R, Kuzmanovic D, Marshall Z, Scheim AI, Mikiki M, Worthington C, Millson MP: The ethics of community-based research with people who use drugs: results of a scoping review. BMC Medical ethics 2016, 17.

Stacciarini JM: A review of community-based participatory research: a promising approach to address depression among Latinos? Issues in Mental Health Nursing 2009, 30(12):751-757.

Stacciarini JM, Shattell MM, Coady M, Wiens B: Review: Community-based participatory research approach to address mental health in minority populations. Community mental health journal 2011, 47(5):489-497.

Strnadova I, Walmsley J: Peer-reviewed articles on inclusive research: Do co-researchers with intellectual disabilities have a voice? Journal of applied research in intellectual disabilities 2018, 31:132-141.

Tamariz L, Medina H, Taylor J, Carrasquillo O, Kobetz E, Palacio A: Are Research Ethics Committees Prepared for Community-Based Participatory Research? Journal of empirical research on human research ethics 2015, 10:488-495.

Tindana P, de Vries J, Campbell M, Littler K, Seeley J, Marshall P, Troyer J, Ogundipe M, Alibu VP, Yakubu A et al: Community engagement strategies for genomic studies in Africa: a review of the literature. BMC Medical ethics 2015, 16.

Tremblay MC, Martin DH, Macaulay AC, Pluye P: Can we Build on Social Movement Theories to Develop and Improve Community-Based Participatory Research? A Framework Synthesis Review. American Journal of Community Psychology 2017, 59:333-362.

Tricco AC, Zarin W, Rios P, Nincic V, Khan PA, Ghassemi M, Diaz S, Pham B, Straus SE, Langlois EV: Engaging policy-makers, heath system managers, and policy analysts in the knowledge synthesis process: a scoping review. Implementation science 2018, 13.

Vaughn LM, Jacquez F, Lindquist-Grantz R, Parsons A, Melink K: Immigrants as Research Partners: A Review of Immigrants in Community-Based Participatory Research (CBPR). Journal of immigrant and minority health 2017, 19:1457-1468.

Vaughn LM, Jones JR, Booth E, Burke JG: Concept mapping methodology and community-engaged research: A perfect pairing. Evaluation and program planning 2017, 60:229-237.

Vaughn LM, Wagner E, Jacquez F: A review of community-based participatory research in child health. MCN-The American journal of maternal-child nursing 2013, 38(1):48-53.

Viswanathan M, Ammerman A, Eng E, Garlehner G, Lohr KN, Griffith D, Rhodes S, Samuel-Hodge C, Maty S, Lux L et al: Community-based participatory research: assessing the evidence. Evid Rep Technol Assess (Summ) 2004(99):1-8.

Vollm B, Foster S, Bates P, Huband N: How Best to Engage Users of Forensic Services in Research: Literature Review and Recommendations. International journal of forensic mental health 2017, 16:183-195.

Wilson MG, Lavis JN, Travers R, Rourke SB: Ethical Challenges in Community-Based Participatory Research: A Scoping Review. Qualitative Health Research, 2018, 28(2), 189-199.

Wine O, Ambrose S, Campbell S, Villeneuve PJ, Burns KK, Vargas AO, Team D: Key Components of Collaborative Research in the Context of Environmental Health: A Scoping Review. Journal of research practice 2017, 13.

Young A, Menon D, Street J, Al-Hertani W, Stafinski T: Exploring patient and family involvement in the lifecycle of an orphan drug: a scoping review. orphaned journal of rare diseases 2017, 12.

Yusuf A, Elsabbagh M: At the cross-roads of participatory research and biomarker discovery in autism: the need for empirical data. BMC Medical ethics 2015, 16: 88

A text file (BIBTEX) including the references is published on OSF.

Appendix 6. Reviews that reported on differences and similarities of different research partnership terms and definitions.

Brizay *et al.* (2015) [2] conducted a systematic comparison of the use of different terms and definitions of research partnership approaches (AR, PAR, CBR, CBPR) in HIV-related research. The authors described the historical change in the use of terms, from AR as the oldest approach to PAR, CBR, and CBPR. In addition, the authors described differences and similarities between PAR and AR as well as CBPR and CBR. Dawson et al. (2018) [3] included an overview of differences and similarities in definitions, original, and principles between PPI and CBPR based on research partnerships with black and ethnic minority groups. Greenhalgh *et al.* (2016) [4] compared four models of co-creation (i.e., value of co-creation, experience-based co-design, experience-based co-design, CBPR) used in different disciplines (i.e., business and management, interdisciplinary, computer science, development studies). Five other reviews [5-9] included a description of differences and similarities between different research partnership approaches in their background and/or discussion sections.

List of abbreviations: AR = Action Research; PAR = Participatory Action Research; CBR = Community-based Research; CBPR = Community-based Participatory Research.

References related to appendix 6:

- Brizay U, Golob L, Globerman J, Gogolishvili D, Bird M, Rios-Ellis B, Rourke SB, Heidari S: Community-academic partnerships in HIV-related research: A systematic literature review of theory and practice. Journal of the International AIDS Society 27, 18.

- Dawson S, Campbell SM, Giles SJ, Morris RL, Cheraghi-Sohi S: Black and minority ethnic group involvement in health and social care research: A systematic review. HEALTH EXPECTATIONS 2018, 21:3-22.
- Greenhalgh T, Jackson C, Shaw S, Janamian T: Achieving Research Impact Through Co-creation in Community-Based Health Services: Literature Review and Case Study. MILBANK QUARTERLY 2016, 94:392-429.
- Di Lorito C, Birt L, Pol, F., Csipke E, Gove D, Diaz-Ponce A, Orrell M: A synthesis of the evidence on peer research with potentially vulnerable adults: how this relates to dementia. International JOURNAL OF GERIATRIC PSYCHIATRY 2017, 32:58-67.
- Ehde DM, Wegener ST, Williams RM, Ephraim PL, Stevenson JE, Isenberg PJ, MacKenzie EJ: Developing, Testing, and Sustaining Rehabilitation Interventions Via Participatory Action Research. ARCHIVES OF PHYSICAL MEDICINE AND REHABILITATION 2013, 94:S30-S42.
- Orlowski SK, Lawn S, Venning A, Winsall M, Jones GM, Wyld K, Damarell RA, Antezana G, Schrader G, Smith D et al: Participatory Research as One Piece of the Puzzle: A Systematic Review of Consumer Involvement in Design of Technology-Based Youth Mental Health and Well-Being Interventions. JMIR HUMAN FACTORS, 2:e12.
- Vaughn LM, Jacquez F, Lindquist-Grantz R, Parsons A, Melink K: Immigrants as Research Partners: A Review of Immigrants in Community-Based Participatory Research (CBPR). JOURNAL OF IMMIGRANT AND MINORITY HEALTH 2017, 19:1457-1468.
- Wine O, Ambrose S, Campbell S, Villeneuve PJ, Burns KK, Vargas AO, Team D: Key Components of Collaborative Research in the Context of Environmental Health: A Scoping Review. JOURNAL OF RESEARCH PRACTICE 2017, 13.

Appendix 7: List of identified terms to describe key domains

Principles	Strategies	Outcomes/Impacts
Approach	Approach	Benefits
Attributes	Challenges	Results
Benefits	Characteristic	Findings
Characteristic	Component	Challenges
Components	Element	Outputs
Element	Enabler	Risks
Facilitator	Facilitator or Barrier	Effects
Factor	Factors	
Impact	Inhibitors	
Mechanisms	Ways	
Norms		
Recommendation		
Strategies		
Values		

Note: Principles and strategies were also identified in the text of the included reviews without a specific description.

Appendix 8: Principles: description of reviews that specifically focused on principles of research partnerships

Description

Eight reviews [10-17] used Israel's principles for community-based research [18] as an inclusion criterion and/or as a guide to structure their review. Baines *et al.* [19] identified 13 Patient and Public Involvement (PPI) principles through a systematic review of reviews (n=13) and grey literature (n=88). The authors also conducted a Delphi study to identify "essential" and "desirable" principles for PPI in health and social care services, research, education and regulation across medicine, dentistry and nursing. Mikesell *et al.* [20] conducted a literature review on ethics in community-based participatory research (CBPR). The authors identified the extent to which the Belmont ethical principles for biomedical research (autonomy, beneficence, and justice) were mentioned in the CBPR literature. In addition to the Belmont principles, the authors identified other components of ethics in CBPR, including community collaboration, community significance, community return, and community control. Greenhalgh *et al.* [4] identified principles for research co-creation through a literature review and a case study. These principles included "a system perspective", "the framing of research as a creative enterprise with human experience at its core", and "an emphasis on process". Nwanyanwu *et al.* found the use of 9 principles of community engagement from the Centers for Disease Control and Prevention [22] in 18 articles in ophthalmology and found that all studies used at least one of these principles.

References related to appendix 8:

- Blair T, Minkler M: Participatory Action Research With Older Adults: Key Principles in Practice. GERONTOLOGIST 2009, 49:651-662.
- Chen PG, Diaz N, Lucas G, Rosenthal MS: Dissemination of Results in Community-Based Participatory Research. AMERICAN JOURNAL OF PREVENTIVE MEDICINE 2010, 39:372-378.
- Crabtree CA: A systematic review of the use of community-based participatory research in natural disaster risk reduction A community-based participatory research approach to assist vulnerable communities in developing a disaster preparedness plan to lessen the impact of natural disasters. University of Hawaii; 2014.
- Nwanyanwu K, Grossetta Nardini HK, Shaughness G, Nunez-Smith M, Newman-Casey P-A: Systematic review of community-engaged research in ophthalmology. EXPERT REVIEW OF OPHTHALMOLOGY 2017, 12:233-241.
- Ragavan MI, Thomas K, Medzhitova J, Brewer N, Goodman LA, Bair-Merritt M: A systematic review of community-based research interventions for domestic violence survivors. PSYCHOLOGY OF VIOLENCE 2019 9(2), 139–155.
- Stacciarini JM: A review of community-based participatory research: a promising approach to address depression among Latinos? ISSUES IN MENTAL HEALTH NURSING 2009, 30:751-757.
- Stacciarini JM, Shattell MM, Coady M, Wiens B: Review: Community-based participatory research approach to address mental health in minority populations. COMMUNITY MENTAL HEALTH JOURNAL 2011, 47(5):489-497.
- Yusuf A, Elsabbagh M: At the cross-roads of participatory research and biomarker discovery in autism: the need for empirical data. BMC MEDICAL ETHICS 2015, 16.
- Israel BA, Schulz AJ, Parker EA, Becker AB: Review of community-based research: assessing partnership approaches to improve public health. ANNU REV PUBLIC HEALTH 1998, 19:173-202.
- Baines RL, de Bere SR: Optimizing patient and public involvement (PPI): Identifying its "essential" and "desirable" principles using a systematic review and modified Delphi methodology. HEALTH EXPECTATIONS 2018, 21:327-335.
- Mikesell L, Bromley E, Khodyakov D: Ethical Community-Engaged Research: A Literature Review. AMERICAN JOURNAL OF PUBLIC HEALTH 2013, 103:E7-E14.

Appendix 9: Strategies: description of the reviews including the highest number of different strategies.

Description

Three Canadian reviews [23-25] reported the highest number of different strategies. Salsberg *et al.* [24] identified key strategies for successful participatory research (PR) partnership approaches and published summarizing strategies and related examples for developing PR partnerships. The authors distinguished between “the most frequently mentioned strategies” and “less frequently mentioned strategies”. Tricco *et al.* [25] summarized their findings in an engagement strategy framework including *stakeholders’ roles* (e.g., key informant, advisory group, steering group), *engagement methods* (e.g., meetings, surveys, Delphi), and *engagement mode* (e.g., in-person, telephone, email). The roles, methods, and modes were also specified for different phases in the research process (conceptualization and design, search and data collection, data synthesis and interpretation, and knowledge dissemination and application). Camden *et al.* [23] conducted a thematic analysis of strategies for stakeholder engagement in rehabilitation research. The authors included a qualitative description of identified strategies as well as a summarizing table including key strategies (e.g., formal and informal training, payment of stakeholders, use of a variety of media and materials to facilitate engagement and understanding).

References related to appendix 9:

- Camden C, Shikako-Thomas K, Nguyen T, Graham E, Thomas A, Sprung J, Morris C, Russell DJ: Engaging stakeholders in rehabilitation research: a scoping review of strategies used in partnerships and evaluation of impacts. DISABILITY AND REHABILITATION 2015, 37:1390-1400.
- Salsberg J, Parry D, Pluye P, Macridis S, Herbert CP, Macaulay AC: Successful strategies to engage research partners for translating evidence into action in community health: A critical review. JOURNAL OF ENVIRONMENTAL AND PUBLIC HEALTH 2015, 2015.
- Tricco AC, Zarin W, Rios P, Nincic V, Khan PA, Ghassemi M, Diaz S, Pham B, Straus SE, Langlois EV: Engaging policy-makers, heath system managers, and policy analysts in the knowledge synthesis process: a scoping review. IMPLEMENTATION SCIENCE 2018, 13

Appendix 10: Outcomes and Impacts: Description of highlighted reviews on outcomes and/or impacts of research partnerships.

Highlighted reviews

Brett *et al.* [26, 27] published two reviews on the impacts of PPI on researchers, stakeholders, communities, and the research process in the field of health and social science. Both reviews provided a detailed qualitative description of potentially beneficial and challenging impacts. Bush *et al.* [28] conducted a systematic review on the additional benefits of organizational participatory research compared to traditional research approaches using quantitative and qualitative methodologies. Besides the identification of types of additional benefits, the authors investigated factors (type of participation, year of publication, initiation by the organization, duration) associated with those additional benefits. Of these factors, only the initiation by the organization was significantly associated with additional benefits indicating benefits may be expected when the organization (stakeholder) initiated the partnership. This finding is in line with the review of Cook (2008) [29] who found that CBPR projects initiated by stakeholders (community or governmental agencies) are more likely to result in community changes or action. However, Gagliardi *et al.* [30] did not find any association between outcomes and initiation of IKT partnerships. Jagosh *et al.* [31] used the Context-Mechanism-Outcome (CMO) framework to describe and explain the outcomes of research partnerships. The authors included a variety of examples to illustrate how and why outcomes (beneficial and challenging) may occur.

References related to appendix 10

- Brett J, Staniszewska S, Mockford C, Herron-Marx S, Hughes J, Tysall C, Suleman R: A Systematic Review of the Impact of Patient and Public Involvement on Service Users, Researchers and Communities. PATIENT-PATIENT CENTERED OUTCOMES RESEARCH 2014, 7:387-395.
- Brett J, Staniszewska S, Mockford C, Herron-Marx S, Hughes J, Tysall C, Suleman R: Mapping the impact of patient and public involvement on health and social care research: a systematic review. HEALTH EXPECTATIONS 2014, 17:637-650.
- Bush PL, Pluye P, Loignon C, Granikov V, Wright MT, Pelletier J-F, Bartlett-Esquilant G, Macaulay AC, Haggerty J, Parry S et al: Organizational participatory research: a systematic mixed studies review exposing its extra benefits and the key factors associated with them. IMPLEMENTATION SCIENCE 2017, 12.
- Cook WK: Integrating research and action: a systematic review of community-based participatory research to address health disparities in environmental and occupational health in the USA. JOURNAL OF EPIDEMIOLOGY AND COMMUNITY HEALTH 2008, 62:668-676.
- Gagliardi AR, Berta W, Kothari A, Boyko J, Urquhart R: Integrated knowledge translation (IKT) in health care: a scoping review. IMPLEMENTATION SCIENCE 2016, 11.
- Jagosh J, Macaulay AC, Pluye P, Salsberg J, Bush PL, Henderson J, Sirett E, Wong G, Cargo M, Herbert CP et al: Uncovering the Benefits of Participatory Research: Implications of a Realist Review for Health Research and Practice. MILBANK QUARTERLY 2012, 910:311-346

References

1. Hoekstra F, Mrklas KJ, Sibley KM, Nguyen T, Vis-Dunbar M, Neilson CJ, Crockett LK, Gainforth HL, Graham ID: **A review protocol on research partnerships: a Coordinated Multicenter Team approach**. *SYSTEMATIC REVIEWS* 2018, **7**(1):217.
2. Brizay U, Golob L, Globerman J, Gogolishvili D, Bird M, Rios-Ellis B, Rourke SB, Heidari S: **Community-academic partnerships in HIV-related research: A systematic literature review of theory and practice**. *JOURNAL OF THE INTERNATIONAL AIDS SOCIETY* 2015, **18**.
3. Dawson S, Campbell SM, Giles SJ, Morris RL, Cheraghi-Sohi S: **Black and minority ethnic group involvement in health and social care research: A systematic review**. *HEALTH EXPECTATIONS* 2018, **21**:3-22.
4. Greenhalgh T, Jackson C, Shaw S, Janamian T: **Achieving Research Impact Through Co-creation in Community-Based Health Services: Literature Review and Case Study**. *MILBANK QUARTERLY* 2016, **94**:392-429.
5. Di Lorito C, Birt L, Pol, F., Csipke E, Gove D, Diaz-Ponce A, Orrell M: **A synthesis of the evidence on peer research with potentially vulnerable adults: how this relates to dementia**. *INTERNATIONAL JOURNAL OF GERIATRIC PSYCHIATRY* 2017, **32**:58-67.
6. Ehde DM, Wegener ST, Williams RM, Ephraim PL, Stevenson JE, Isenberg PJ, MacKenzie EJ: **Developing, Testing, and Sustaining Rehabilitation Interventions Via Participatory Action Research**. *ARCHIVES OF PHYSICAL MEDICINE AND REHABILITATION* 2013, **94**:S30-S42.
7. Orlowski SK, Lawn S, Venning A, Winsall M, Jones GM, Wyld K, Damarell RA, Antezana G, Schrader G, Smith D *et al*: **Participatory Research as One Piece of the Puzzle: A Systematic Review of Consumer Involvement in Design of Technology-Based Youth Mental Health and Well-Being Interventions**. *JMIR HUMAN FACTORS* 2015, **2**:e12.
8. Vaughn LM, Jacquez F, Lindquist-Grantz R, Parsons A, Melink K: **Immigrants as Research Partners: A Review of Immigrants in Community-Based Participatory Research (CBPR)**. *JOURNAL OF IMMIGRANT AND MINORITY HEALTH* 2017, **19**:1457-1468.
9. Wine O, Ambrose S, Campbell S, Villeneuve PJ, Burns KK, Vargas AO, Team D: **Key Components of Collaborative Research in the Context of Environmental Health: A Scoping Review**. *JOURNAL OF RESEARCH PRACTICE* 2017, **13**.
10. Blair T, Minkler M: **Participatory Action Research With Older Adults: Key Principles in Practice**. *GERONTOLOGIST* 2009, **49**:651-662.
11. Chen PG, Diaz N, Lucas G, Rosenthal MS: **Dissemination of Results in Community-Based Participatory Research**. *AMERICAN JOURNAL OF PREVENTIVE MEDICINE* 2010, **39**:372-378.
12. Crabtree CA: **A systematic review of the use of community-based participatory research in natural disaster risk reduction** *A community-based participatory research approach to assist vulnerable communities in developing a disaster preparedness plan to lessen the impact of natural disasters.* University of Hawaii; 2014.

13. Nwanyanwu K, Grossetta Nardini HK, Shaughness G, Nunez-Smith M, Newman-Casey P-A: **Systematic review of community-engaged research in ophthalmology**. *EXPERT REVIEW OF OPHTHALMOLOGY* 2017, **12**:233-241.
14. Ragavan MI, Thomas K, Medzhitova J, Brewer N, Goodman LA, Bair-Merritt M: **A systematic review of community-based research interventions for domestic violence survivors**. *PSYCHOLOGY OF VIOLENCE* 2019 9(2), 139–155.
15. Stacciarini JM: **A review of community-based participatory research: a promising approach to address depression among Latinos?** *Issues in Mental Health Nursing* 2009, **30**:751-757.
16. Stacciarini JM, Shattell MM, Coady M, Wiens B: **Review: Community-based participatory research approach to address mental health in minority populations**. *Community mental health journal* 2011, **47**(5):489-497.
17. Yusuf A, Elsabbagh M: **At the cross-roads of participatory research and biomarker discovery in autism: the need for empirical data**. *BMC MEDICAL ETHICS* 2015, **16**.
18. Israel BA, Schulz AJ, Parker EA, Becker AB: **Review of community-based research: assessing partnership approaches to improve public health**. *Annu Rev Public Health* 1998, **19**:173-202.
19. Baines RL, de Bere SR: **Optimizing patient and public involvement (PPI): Identifying its "essential" and "desirable" principles using a systematic review and modified Delphi methodology**. *HEALTH EXPECTATIONS* 2018, **21**:327-335.
20. Mikesell L, Bromley E, Khodyakov D: **Ethical Community-Engaged Research: A Literature Review**. *AMERICAN JOURNAL OF PUBLIC HEALTH* 2013, **103**:E7-E14.
21. Nwanyanwu KH, Grossetta Nardini HK, Shaughness G, Nunez-Smith M, Newman-Casey PA: **Systematic Review of Community-Engaged Research in Ophthalmology**. *EXPERT REV OPHTHALMOL* 2017, **12**(3):233-241.
22. Centers for Disease Control and Prevention: **Principles of community engagement**. 2011.
23. Camden C, Shikako-Thomas K, Nguyen T, Graham E, Thomas A, Sprung J, Morris C, Russell DJ: **Engaging stakeholders in rehabilitation research: a scoping review of strategies used in partnerships and evaluation of impacts**. *DISABILITY AND REHABILITATION* 2015, **37**:1390-1400.
24. Salsberg J, Parry D, Pluye P, Macridis S, Herbert CP, Macaulay AC: **Successful strategies to engage research partners for translating evidence into action in community health: A critical review**. *JOURNAL OF ENVIRONMENTAL AND PUBLIC HEALTH* 2015, **2015**.
25. Tricco AC, Zarin W, Rios P, Nincic V, Khan PA, Ghassemi M, Diaz S, Pham B, Straus SE, Langlois EV: **Engaging policy-makers, heath system managers, and policy analysts in the knowledge synthesis process: a scoping review**. *IMPLEMENTATION SCIENCE* 2018, **13**.
26. Brett J, Staniszewska S, Mockford C, Herron-Marx S, Hughes J, Tysall C, Suleman R: **A Systematic Review of the Impact of Patient and Public Involvement on Service Users,**

Researchers and Communities. *PATIENT-PATIENT CENTERED OUTCOMES RESEARCH* 2014, **7**:387-395.

27. Brett J, Staniszewska S, Mockford C, Herron-Marx S, Hughes J, Tysall C, Suleman R: **Mapping the impact of patient and public involvement on health and social care research: a systematic review**. *HEALTH EXPECTATIONS* 2014, **17**:637-650.
28. Bush PL, Pluye P, Loignon C, Granikov V, Wright MT, Pelletier J-F, Bartlett-Esquilant G, Macaulay AC, Haggerty J, Parry S *et al*: **Organizational participatory research: a systematic mixed studies review exposing its extra benefits and the key factors associated with them**. *IMPLEMENTATION SCIENCE* 2017, **12**.
29. Cook WK: **Integrating research and action: a systematic review of community-based participatory research to address health disparities in environmental and occupational health in the USA.** *Journal of epidemiology and community health* 2008, **62**:668-676.
30. Gagliardi AR, Berta W, Kothari A, Boyko J, Urquhart R: **Integrated knowledge translation (IKT) in health care: a scoping review**. *IMPLEMENTATION SCIENCE* 2016, **11**.
31. Jagosh J, Macaulay AC, Pluye P, Salsberg J, Bush PL, Henderson J, Sirett E, Wong G, Cargo M, Herbert CP *et al*: **Uncovering the Benefits of Participatory Research: Implications of a Realist Review for Health Research and Practice**. *MILBANK QUARTERLY* 2012, **90**:311-346.

PRISMA 2009 Checklist

Section/topic	#	Checklist item	Reported on page #
**TITLE**			
Title	1	Identify the report as a systematic review, meta-analysis, or both.	Page 1 – identify as review of reviews.
**ABSTRACT**			
Structured summary	2	Provide a structured summary including, as applicable: background; objectives; data sources; study eligibility criteria, participants, and interventions; study appraisal and synthesis methods; results; limitations; conclusions and implications of key findings; systematic review registration number.	Page 4-5
**INTRODUCTION**			
Rationale	3	Describe the rationale for the review in the context of what is already known.	Page 6 + Protocol paper
Objectives	4	Provide an explicit statement of questions being addressed with reference to participants, interventions, comparisons, outcomes, and study design (PICOS).	Page 7 – line 139-142 + Table 1.
**METHODS**			
Protocol and registration	5	Indicate if a review protocol exists, if and where it can be accessed (e.g., Web address), and, if available, provide registration information including registration number.	Page 7-8 – line 151-153
Eligibility criteria	6	Specify study characteristics (e.g., PICOS, length of follow-up) and report characteristics (e.g., years considered, language, publication status) used as criteria for eligibility, giving rationale.	Table 1
Information sources	7	Describe all information sources (e.g., databases with dates of coverage, contact with study authors to identify additional studies) in the search and date last searched.	Page 8 – line 156-160
Search	8	Present full electronic search strategy for at least one database, including any limits used, such that it could be repeated.	Protocol paper + OSF Table I.

PRISMA 2009 Checklist

Study selection	9	State the process for selecting studies (i.e., screening, eligibility, included in systematic review, and, if applicable, included in the meta-analysis).	Page 9-10, lines 190-204
Data collection process	10	Describe method of data extraction from reports (e.g., piloted forms, independently, in duplicate) and any processes for obtaining and confirming data from investigators.	Page 10-12.
Data items	11	List and define all variables for which data were sought (e.g., PICOS, funding sources) and any assumptions and simplifications made.	Appendix 1
Risk of bias in individual studies	12	Describe methods used for assessing risk of bias of individual studies (including specification of whether this was done at the study or outcome level), and how this information is to be used in any data synthesis.	Page 13 – Lines 271-275
Summary measures	13	State the principal summary measures (e.g., risk ratio, difference in means).	n/a
Synthesis of results	14	Describe the methods of handling data and combining results of studies, if done, including measures of consistency (e.g., I^2) for each meta-analysis.	n/a

Section/topic	#	Checklist item	Reported on page #
Risk of bias across studies	15	Specify any assessment of risk of bias that may affect the cumulative evidence (e.g., publication bias, selective reporting within studies).	n/a
Additional analyses	16	Describe methods of additional analyses (e.g., sensitivity or subgroup analyses, meta-regression), if done, indicating which were pre-specified.	n/a
**RESULTS**			
Study selection	17	Give numbers of studies screened, assessed for eligibility, and included in the review, with reasons for exclusions at each stage, ideally with a flow diagram.	Figure 1
Study characteristics	18	For each study, present characteristics for which data were extracted (e.g., study size, PICOS, follow-up period) and provide the citations.	Table 2 + OSF
Risk of bias within studies	19	Present data on risk of bias of each study and, if available, any outcome level assessment (see item 12).	n/a
Results of individual studies	20	For all outcomes considered (benefits or harms), present, for each study: (a) simple summary data for each intervention group (b) effect estimates and confidence intervals, ideally with a forest plot.	Tables 3, 5-7 + OSF
Synthesis of results	21	Present results of each meta-analysis done, including confidence intervals and measures of consistency.	
Risk of bias across studies	22	Present results of any assessment of risk of bias across studies (see Item 15).	Tables 3, 5-7

PRISMA 2009 Checklist

Additional analysis	23	Give results of additional analyses, if done (e.g., sensitivity or subgroup analyses, meta-regression [see Item 16]).	n/a
**DISCUSSION**			
Summary of evidence	24	Summarize the main findings including the strength of evidence for each main outcome; consider their relevance to key groups (e.g., healthcare providers, users, and policy makers).	Page 23 – lines 494-497
Limitations	25	Discuss limitations at study and outcome level (e.g., risk of bias), and at review-level (e.g., incomplete retrieval of identified research, reporting bias).	Page 25-27
Conclusions	26	Provide a general interpretation of the results in the context of other evidence, and implications for future research.	Page 28-29
**FUNDING**			
Funding	27	Describe sources of funding for the systematic review and other support (e.g., supply of data); role of funders for the systematic review.	Page 31

From: Moher D, Liberati A, Tetzlaff J, Altman DG, The PRISMA Group (2009). Preferred Reporting Items for Systematic Reviews and Meta-Analyses: The PRISMA Statement. PLoS Med 6(6): e1000097. doi:10.1371/journal.pmed1000097

For more information, visit: **www.prisma-statement.org**.
