# Supplementary material for: A review of reviews on principles, strategies, outcomes and impacts of research partnerships approaches: a first step in synthesising the research partnership literature
Source: Health Res Policy Syst. 2020 May 25;18:51. doi: 10.1186/s12961-020-0544-9 (PMC7249434; doi:10.1186/s12961-020-0544-9)
Supplement: Supplementary file 1 — Additional file 1. [file 12961_2020_544_MOESM1_ESM.zip › 12961_2020_544_MOESM1_ESM/Explanation to proofs.docx]

Dear Editor,

Please find our responses to the queries.

Please note that I have attached the following files:

- A PDF version of our Supplementary Files, in which all appendices are combined into one PDF-document. Please note that I have updated the reference of our Open Science Framework page, as this page is no publicly available including a DOI. I did not make any other changes to the content of the supplementary files.
- Response to Query 4 is attached in file titled “Table 2 and 3 – references”
- I included a few comments/corrections in the PDF of the proofs.

Please let me know if you have any questions.

Thank you so much.

Take care,

Femke Hoekstra
